# Principles and practice of determining metal–protein affinities

**DOI:** 10.1042/BCJ20200838

**Published:** 2021-03-10

**Authors:** Tessa R. Young, Zhiguang Xiao

**Affiliations:** 1 Department of Biosciences, Durham University, Durham, U.K.; 2 Department of Chemistry, Durham University, Durham, U.K.; 3 Melbourne Dementia Research Centre, Florey Institute of Neuroscience and Mental Health, The University of Melbourne, Parkville, VIC, Australia; 4 School of Chemistry and Bio21 Molecular Science and Biotechnology Institute, The University of Melbourne, Parkville, VIC, Australia

**Keywords:** dissociation constant, metal probe, metal–protein affinity, metalloprotein, spectroscopic probe, transition metals

## Abstract

Metal ions play many critical roles in biology, as structural and catalytic cofactors, and as cell regulatory and signalling elements. The metal–protein affinity, expressed conveniently by the metal dissociation constant, *K*_D_, describes the thermodynamic strength of a metal–protein interaction and is a key parameter that can be used, for example, to understand how proteins may acquire metals in a cell and to identify dynamic elements (e.g. cofactor binding, changing metal availabilities) which regulate protein metalation *in vivo*. Here, we outline the fundamental principles and practical considerations that are key to the reliable quantification of metal–protein affinities. We review a selection of spectroscopic probes which can be used to determine protein affinities for essential biological transition metals (including Mn(II), Fe(II), Co(II), Ni(II), Cu(I), Cu(II) and Zn(II)) and, using selected examples, demonstrate how rational probe selection combined with prudent experimental design can be applied to determine accurate *K*_D_ values.

## Introduction

Metal–protein interactions have played many critical roles in biology since life first evolved. Around one third of all proteins and nearly half of all enzymes require a metal cofactor for their functions [1,2], but non-essential metals can be toxic and even the essential metals, if not handled properly, can be detrimental [3]. Evaluation and understanding of the acquisition of metals by competing protein sites, in healthy or diseased cells, is a major focus in the fascinating field of Metallomics. The thermodynamic affinity of a metal–protein interaction, expressed by the metal dissociation constant, *K*_D_, is a key parameter for such evaluation and understanding in a biological context. Innovative new research has quantified the thermodynamic availability of common transition metals inside a cell [4,5], making it possible to predict *in vivo* metal occupancy of proteins using their *in vitro* affinity data as input [6]. However, the accuracy of these calculations relies on the accurate quantification of such affinity data from *in vitro* experiments which is not a trivial matter and requires a combination of meticulous sample preparation, judicious experimental design, careful execution of experiments with appropriate controls, and adequate analysis and processing of the experimental data. Mishandling of these processes may lead to large errors in the determined affinity values, as exemplified by the highly dispersed metal affinity data reported in the literature for many proteins including, notoriously, Cu(I)-binding proteins, amyloid β peptides and zinc figure peptides whose reported affinities are scattered by 2–4 (or even >10 in certain cases) orders of magnitude [7–9]. This review outlines the fundamental principles and practical considerations which are key to accurate evaluation of metal–protein affinities, including the conditions under which direct titration or ligand competition approaches can be reliably employed (some other general aspects on determining metal affinities are covered elsewhere [10–12]). Furthermore, we assess a suite of spectroscopic ligand probes which can be used readily and conveniently to quantify a diverse range of biologically significant metal-binding interactions.

## Metal–protein affinity and the conditional dissociation constant

A metal ion, M_aq_, and ligand, L, are Lewis acid and base, respectively, and may react to form a metal complex, ML, according to equation 1a with *K*_ex_ and *K*_A(L)_ defined by equation 1b:

(1a) $${\text{M}}_{\mathrm{aq}}+\text{L }\;⇌\;\mathrm{ML}\;+{\;}^{\prime }x{\text{H}}_{2}O^{\prime }\;$$

(1b) $$K_{\mathrm{ex}}=\;\frac{[\text{ML}]{\left[{\text{H}}_{2}\text{O}\right]}^{x}}{\left[{\text{M}}_{\mathrm{aq}}][\text{L}\right]}\;=\;K_{A(L)}\;[{\text{H}}_{2}\text{O}{]}^{x}$$

where *x* is the number of coordinating H_2_O ligands in M_aq_ and [ML] should be read as the equilibrium concentration of the metal–ligand complex, ML, and likewise for all other species inside a bracket hereafter. Since *K*_ex_ is a constant and [H_2_O] is virtually unchanged (given [M_aq_] ≪ [H_2_O] = 55.5 M in aqueous solution), the association constant *K*_A(L)_ for ML defined by equation 1b is also a constant.

The first quantitative determination of a *K*_A(L)_ for a ML complex started about 80 years ago when the then newly developed glass electrode and pH metre enabled Jannik Bjerrum to develop the first potentiometric titration method to evaluate the equilibrium 1a quantitatively by introducing a proton competition for the metal ligand into the system which is described by equation 2 [13]:

(2) $${\text{M}}_{\mathrm{aq}}\;+\;\mathrm{HL}⇌\text{ML }\;+\;H$$

Potentiometry is still the gold standard, even today, for the accurate determination of association constants defined by equation 1b. However, this method is subject to a strict limitation: the equilibria of all exchangeable protons in the system must be accounted for [14,15]. This remains an impossible challenge for metal–protein interactions because: (i) the contributions of large numbers of exchangeable protons from multiple amino-acid sidechains cannot be resolved by this method; and (ii) protein stabilities are sensitive to pH, with structures commonly modified or even unfolded at extremes. Consequently, the association constant, *K*_A(P)_, of a metal–protein complex, MP, cannot be determined by potentiometric titration.

In protein biochemistry, the stability of a metal–protein complex is most frequently described by the dissociation constant, *K*_D(P)_ (i.e. the inverse of the association constant *K*_A(P)_), defined by equation 3:

(3) $$\text{MP }\;⇌\;{\text{M}}_{\mathrm{aq}}+\text{P }\;\;\;\;\;\;\;\;K_{D(P)}=\;\frac{\left[{\text{M}}_{\mathrm{aq}}][\text{P}\right]}{\left[\text{MP}\right]}\;$$

*K*_D(P)_ is defined as the concentration of hydrated metal, [M_aq_] (frequently referred to as free metal), in solution when exactly half of the protein is metal-occupied (i.e. *K*_D(P)_ = [M_aq_] when [P] = [MP]). This inverse parameter has more tangible meaning in a biological context since it can be directly compared with the availability of metal ions in a cell [5]. Metal availabilities may also be expressed by p[M] = −log [M_aq_], analogous to expressing free proton concentrations by pH. Notably, while potentiometry assesses the competition between metal M and proton H for binding L over a broad range of pH and explicitly illustrates the metal and proton stoichiometries of each component involved in equation 2 [15], alternative methods to analyse the equilibrium of equation 3 (see sections ‘Determination of metal–protein affinities via direct metal titration’ and ‘Determination of metal–protein affinities via competition experiments’) cannot distinguish between different protonation states of the MP species. Instead, they deal with ensembles of species present at the equilibrium. Consequently, a *K*_D(P)_ value is a conditional constant that is pH-dependent, that could be a weighted-average value of many species in rapid equilibrium and that must always be reported as corresponding to a specific pH.

## Determination of metal–protein affinities via direct metal titration

### Direct metal titration and practical challenges

The direct metal titration method is defined here as an experimental condition where H_2_O molecules are the only ligands that compete with the protein for metal-binding according to the *K*_D_ definition of equation 3. Under this condition, [M]_tot_ = [MP] + [M_aq_] and the fraction of total metal partitioned to the protein is given by equation 4:

(4) $$\frac{\left[\text{MP}\right]}{{\left[\text{M}\right]}_{\mathrm{tot}}}\;=\;\frac{1}{1\;+\;K_{D(P)}/[\text{P}]}\;$$

**Figure 1.**
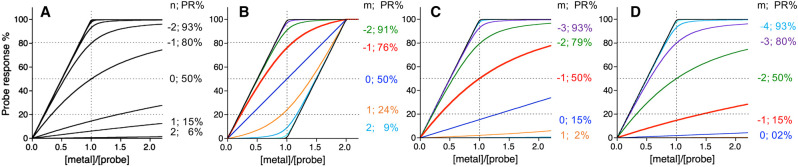
Sensitivity of probe response to measured equilibrium. Simulated probe responses for titration of metal ions into: (**a**) a solution containing a metal probe P (10 µM) with *K*_D(P)_ = 5 × 10*^n^* (*n* = 0, ±1, ±2,…) µM; (**b**) a solution containing an equimolar concentration of metal probe P and competing ligand L (each 10 µM; the case 3 in Table 1) where $m=\;\log\;K_{D(P)}/K_{D(L)}$; (**c**) the same as (**b**) but [L] = 55 µM (the case 2 in Table 1); (**d**) the same as (**b**) but [L] = 505 µM (the case 1 in Table 1). In each case, the dashed lines mark the 20%, 50% and 80% probe responses. The probe response (PR%) at [metal]/[probe] = 1 for each titration curve with specific value *n* in (**a**) or *m* in (**b**–**d**) is also indicated.

It is apparent from this relationship that, if $K_{D(P)}\gg [P]$, then $([\text{MP}]/{\left[\text{M}\right]}_{\mathrm{tot}})\;\to 0$ and the affinity is too weak to be determined under the condition (essentially no metal-binding is observed) and if $K_{D(P)}\ll [P]$, then $([\text{MP}]/{\left[\text{M}\right]}_{\mathrm{tot}})\to 1$ and the affinity is too tight to be determined under the condition (essentially stoichiometric metal-binding is observed). Indeed, only when $K_{D(P)}∼[P]$ will the changes in *K*_D(P)_ be sensitive to the changes in metal partitioning $\left([\text{MP}]/{\left[\text{M}\right]}_{\mathrm{tot}}\right)$ that can be measured experimentally. This concept is illustrated by Figure 1A which simulates the titration of metal into a solution of protein where there is a response readout for M-binding to P. When $K_{D(P)}=[P]$, then the binding isotherm passes the 50% full probe response point at one equivalent of metal titration (i.e.$\;[\text{MP}]/[\text{M}{]}_{\mathrm{tot}}=0.5$ when [P]_tot_ = [M]_tot_ in solution, noting [P] = 0.5 [P]_tot_ under this condition; see Figure 1A). When *K*_D(P)_ is varied by consecutive orders of magnitude (i.e. log *K*_D(P)_ = log (0.5 [P]_tot_) ± *n* (*n* = 1, 2, …)), the binding isotherms diverge sensitively within the 20–80% response range (i.e. the range where$\;[\text{MP}]/[\text{M}{]}_{\mathrm{tot}}\;=0.2-0.8$ when [P]_tot_ = [M]_tot_) but converge rapidly outside this range (Figure 1A). Therefore, the high-affinity limit of quantifying a metal–protein interaction may be set to ∼80% of the full response upon titration of one equivalent of metal (Figure 1A) for the direct metal titration method: This corresponds to an experimentally accessible *K*_D(P)_ limit ∼20 times below the experimental protein concentration (i.e. *K*_D(P)_ ≥ 0.05 [P]tot) [16].

**Table 1.** Optimisation of conditions for determining metal–protein affinities at [M]_tot_ = 10 µM^1^

No	log*K*_ex_^2^	[P]_tot_^3^ (µM)	[MP]^4^ (µM)	[ML] or [ML_2_]^4^ (µM)	[L]_tot_ (1 : 1)^4^ (µM)	[L]_tot_ (1 : 2)^4^ (µM)	[MP]/[P]_tot_	[ML]/[L]_tot_ or 2[ML_2_]/[L]_tot_
1	−2.0	10	5.0	5.0	505	—	0.5	0.01
2	−1.0	10	5.0	5.0	55	—	0.5	0.09
3	0.0	10	5.0	5.0	10	—	0.5	0.5
4	1.0	55	5.0	5.0	10	—	0.09	0.5
5	1.0	10	5.0	5.0	—	717	0.5	0.01
6	2.0	10	5.0	5.0	—	234	0.5	0.04
7	3.0	10	5.0	5.0	—	81	0.5	0.12
8	4.0	10	5.0	5.0	—	32	0.5	0.3
9^5^	4.7	10	5.0	5.0	—	20	0.5	0.5
		↓ 16	↓ 5.0	↓ 5.0		↓ 25	↓ 0.3	↓ 0.4
10	6.0	230	5.0	5.0	—	25	0.02	0.4

1 [M]_tot_ is decided by the detection sensitivity. Selection of [M]_tot_ = 10 µM here is based on an assumption that the response of the selected probe is sensitive enough for a reliable quantification of the metal–probe complex MP in the system, but the [M]_tot_ term may be scaled up or down along with [P]_tot_ and [L]_tot_ according to equations 8a and 8b, respectively. For example, the condition of $\text{log}K_{\mathrm{ex}}=0$ for the stoichiometric competition of case 3 involving a 1 : 1 ML complex remains unchanged with the reaction re-scaling, but $\text{log}K_{\mathrm{ex}}$ for the same stoichiometric reaction of case 9 involved a 1 : 2 ML_2_ complex changes with [M]_tot_ according to $\text{log}K_{\mathrm{ex}}=-\text{log}(2{\left[\text{M}\right]}_{\mathrm{tot}})$ and thus the value $\text{log}K_{\mathrm{ex}}$ = 4.7 applies to the case of [M]_tot_ = 10 µM only;

2 $K_{\mathrm{ex}}=K_{D(P)}/K_{D(L)}$ for equation 7a or $K_{D(P)\;}\beta_{2}$ for equation 7b;

3 [P]_tot_ ≥ [M]_tot_;

4 Calculated via equation 8a or 8b with the condition that $\left([\text{MP}]/{\left[\text{M}\right]}_{\mathrm{tot}}=0.5\right)$;

5 The experiment of case 9 with a molar ratio $\left({\left[\text{L}\right]}_{\mathrm{tot}}/{\left[\text{M}\right]}_{\mathrm{tot}}∼2\right)$ may run the risk of forming some 1 : 1 ML complex and it is generally advisable to set $({\left[\text{L}\right]}_{\mathrm{tot}}/{\left[\text{M}\right]}_{\mathrm{tot}})\geq 2.5$ to ensure ML_2_ complex formation: thus, a new set of conditions for case 9 may be re-set as the arrows suggest.

To allow a best-fit *K*_D(P)_ of the experimental data covering an entire set of the titration data points, equation 3 may be rearranged to equation 5 with incorporation of both [M]_tot_ and [P]_tot_ under the conditions of [M_aq_] = [M]_tot_ − [MP] and [P] = [P]_tot_ − [MP]:

(5) $$[\text{MP}]\;=\;\;\frac{K_{D(P)}+{\left[\text{M}\right]}_{\mathrm{tot}}+{\left[\text{P}\right]}_{\mathrm{tot}}-\sqrt{{\left(K_{D(P)}+{\left[\text{M}\right]}_{\mathrm{tot}}+{\left[\text{P}\right]}_{\mathrm{tot}}\right)}^{2}-4{\left[\text{M}\right]}_{\mathrm{tot}}{\left[\text{P}\right]}_{\mathrm{tot}}}}{2}$$

In theory, a direct metal titration can be used to determine *K*_D(P)_ of a MP complex provided that the above-mentioned conditions can be met, but in practice this can be a difficult challenge for many transition metals because:

- Regarding the condition that *K*_D(P)_ ≥ 0.05 [P]_tot_: Protein concentrations in the micromolar (µM) range are typically required to obtain a measurable metal-binding response: This puts many metal–protein interactions (where *K*_D(P) _< 10^−7 ^M) beyond the tight-binding limit of detection [11].
- Regarding the condition that [M_aq_] = [M]_tot_ − [MP]: While the condition [P] = [P]_tot_ − [MP] can be easily met in most cases, the condition [M_aq_] = [M]_tot_ − [MP] can be challenging. The speciation of ‘unbound’ metal ions (i.e. those not involved in the MP complex) in direct experiments can be ambiguous and often far from 100% in the hydrated form. Many free transition metal ions, when present at µM concentrations in a neutral protein-containing buffer, are vulnerable to attack by adventitious ligands including buffers, salts, hydroxide and adventitious amino-acid ligands of the protein itself and the contributions of such complexes to the overall metal speciation can be substantial but are commonly unappreciated (see section ‘Adventitious metal ligands and the apparent dissociation constant’). Furthermore, some ‘free’ metal ions are unstable at µM concentrations: For example, Cu_aq_^+^ can spontaneously disproportionate into Cu_aq_^2+^ and Cu(0) at acidic pH, or precipitate as insoluble Cu_2_O at neutral and basic pH [17].

While the first condition sets a limit for a detectable *K*_D(P)_ of a particular target via direct metal titration, the second is sometimes impossible to achieve practically and is discussed in detail in section ‘Adventitious metal ligands and the apparent dissociation constant’. However, these two major challenges can be circumvented using a ligand competition approach which is currently the gold standard for determining metal–protein affinities and is detailed in section ‘Determination of metal–protein affinities via competition experiments’. In certain cases, inter-metal competition is also a solution, especially if the free metals are protected with some non-competitive weak ligands (see section ‘Inter-metal competition experiments’).

### Adventitious metal ligands and the apparent dissociation constant

For many transition metals, the condition [M_aq_] = [M]_tot _− [MP] that is required by direct metal titration may be challenged by significant contributions from adventitious ligands, L_ad_, present in the protein solution: Common examples include buffers, salts, reductants and amino-acid sidechains of the protein itself, that may interact with free M_aq_ at high concentrations (≥µM) to generate various additional (often undefined and unaccountable) metal complexes, $\sum [\text{M}({\text{L}}_{\mathrm{ad}})]$, and, as such, equation 4 must be modified to equation 6a:

(6a) $$\frac{\left[\text{MP}\right]}{{\left[\text{M}\right]}_{\mathrm{tot}}}\;=\;\frac{1}{1\;+\;K_{D(P)}\left(1+\;\sum K_{A}[{\text{L}}_{\mathrm{ad}}]\right)/[\text{P}]}$$

Thus, the *K*_D(P)_ term as defined by equation 4 has been modified by a factor of $\left(1+\;\sum K_{A}[{\text{L}}_{\mathrm{ad}}]\right)$, where *K*_A_ are the association constants of the adventitious metal complexes, $\text{M}({\text{L}}_{\mathrm{ad}})$, at the experimental pH and [L_ad_] are the equilibrium concentrations of the adventitious metal ligands. By analogy to the term *K*_D(P)_ in equation 4, equation 6a defines an apparent dissociation constant *K*_D(P)_ (denoted as ^a^*K*_D(P)_) according to equation 6b:

(6b) $${}^{a}K_{D(P)}=K_{D(P)}\left(1+\;\sum K_{A}[{\text{L}}_{\mathrm{ad}}]\right)\;$$

This term is sometimes referred to as a conditional dissociation constant, ^c^*K*_D(P)_, but we recommend that the word ‘conditional’ should be reserved for describing the pH effect on the metal ligand (the protein, P, in this case) and the word ‘apparent’ be reserved for describing the effects of other competing metal ligands on the metal centre.

The ^a^*K*_D(P)_ term, defined by equation 6b, may be regarded as a measure of the sum of the concentrations of all protein-unbound metal-containing species, $\left({\left[\text{M}\right]}_{\mathrm{aq}}+\sum [\text{M}({\text{L}}_{\mathrm{ad}})]\right)$, at 50% metal occupancy of the protein P. Many ^a^*K*_D(P)_ values, defined this way, have been determined by direct metal titration but have been reported simply as (conditional) *K*_D(P)_: This is highly problematic and has led to erroneous *K*_D(P)_ values being propagated in the literature. It can be seen from equation 6b that the condition for ^a^*K*_D(P)_ ∼ *K*_D(P)_ is $\sum K_{A}[{\text{L}}_{\mathrm{ad}}]\ll 1$. However, this condition is often not met, even for a weak-binding buffer with small *K*_A_ since its concentration is relatively high. For example, although Tris buffer has a relatively modest affinity for Cu^2+^ (*K*_D_ ∼ 0.6 mM at pH 7 [18]) a direct titration of Cu^2+^ into protein in the presence of 50 mM Tris at pH 7 will underestimate the conditional protein affinity by almost 2 orders of magnitude (i.e. the term *K*_A_[L_ad_] ∼ 83) if the buffer is not accounted for.

The impacts of adventitious ligands (buffers, reductants, salts, etc.) may be ruled out as negligible if systematic control titrations using the same [P] but with varying [L_ad_] are indistinguishable [19] (i.e. $\sum K_{A}[{\text{L}}_{\mathrm{ad}}]\ll 1$), or may be accounted for if the term $\sum K_{A}[{\text{L}}_{\mathrm{ad}}]$ can be estimated (in such cases the experiment may be broadly classified as a ligand competition between L_ad_ and [P]) [20–22]. However, in many direct metal titrations the impacts of L_ad_ are neither negligible nor accounted for (*K*_A_ and $\sum [{\text{L}}_{\mathrm{ad}}]$ may be unknown, for example) and the resulting ^a^*K*_D(P)_ value may hardly be a true ‘constant’ and may underestimate the conditional metal–protein affinity by many orders of magnitude [7,9,11,23].

### Illustrative examples of metal affinity determination by direct metal titration

Although it can be difficult to determine reliably a (pH-conditional) *K*_D(P)_ for transition metal-binding to a protein by direct metal titration due to the two challenges discussed above, it is possible, when *K*_D(P)_ ≥ 0.05 [P]_tot_, for simple metal ligands and some small peptides with careful control of the experimental conditions. For examples, the Cu(II) *K*_D_ (= 0.38 µM for the fully deprotonated ligand) of a Monovalent Copper Ligand, MCL-1, was readily determined by direct Cu^2+^ titration of MCL-1 at 50 µM in a pH 5.0 buffer, which increased the complex dissociation (due to the protonation of MCL-1, pK_a_ ∼ 7.1) and suppressed Cu^2+^ hydrolysis at high µM concentrations [24]. The weak Ni(II) affinities of several zinc finger peptides (*K*_D_ ∼ 3 µM) were determined readily by direct metal titration, but the corresponding Co(II) affinities at *K*_D_ ∼ 3 nM are too tight to be determined reliably by direct metal titration of the peptides at 10 s of µM [23]. In one of our best efforts to determine the Cu(II) affinity of a sensitive fluorescent peptide probe (DP1, see section ‘Dansylated peptides (DP1–4)’) by direct Cu^2+^ titration, we tackled the two challenges discussed above by: (i) employing the lowest detectable DP1 concentration (0.2 µM) to ensure a detectable dissociation of the target Cu^II^–DP1 complex; and (ii) conducting the experiments in a carefully controlled pH buffer of low Cu(II) affinity (Mops, pH 7.4) at a limiting concentration of only 0.5 mM without addition of any salts to ensure $\sum K_{A}[{\text{L}}_{\mathrm{ad}}]\ll 1$. In this case, the apparent affinity estimated by direct metal titration (with the experimental data fitted to equation 5) was ^a^*K*_D(P)_ = 10^−8.0^ M which agreed with the conditional *K*_D(P)_ = 10^−8.1 ^M determined by competition with the standard ligand glycine [19]. However, our same efforts to determine the Cu(II) affinity of a similar fluorescent peptide, Aβ16-WWA, allowed estimation of only a limiting ^a^*K*_D(P)_ < 10^−9.1^ M by direct metal titration but this value was still significantly larger (weaker in affinity) than the conditional *K*_D(P)_ = 10^−9.8 ^M that we determined by ligand competition using two independent affinity standards [25]. Apparently, this sub-nanomolar (nM) affinity was too tight to be accessible by direct metal titration, even with the lowest possible experimental peptide concentration of just 0.2 µM. Thus, only for a limited number of cases where a metal–protein interaction is relatively weak and can be sensitively detected (i.e. enabling the experiment to be conducted at [P] ∼ *K*_D(P)_), may a direct metal titration provide an accurate estimation of *K*_D(P)_ under the condition of $\sum K_{A}[{\text{L}}_{\mathrm{ad}}]\ll 1$.

Notoriously, the Cu(II) affinities of Aβ peptides were underestimated by 2–3 orders of magnitude in early years by direct metal titration but consolidated in later years by ligand competition [7,25,26]. Likewise, the Co(II) affinities of many zinc figure peptides were determined by direct metal titration with the convenience and advantage of the spectroscopic properties offered by this open d-shell metal, but the reported data are highly scattered and have also been underestimated, typically by 2–3 orders of magnitude, when compared with those data acquired via more stringent methods such as ligand competition or potentiometry [9,23]. These examples illustrate how use of the direct metal titration method to determine *K*_D(P)_ values can be challenging and problematic, particularly for tight metal–protein interactions. Even where measurable dissociation is observed, estimates of *K*_D(P)_ may be affected by contributions from adventitious ligands (including buffers, salts and reductants, see section ‘Adventitious metal ligands and the apparent dissociation constant’ discussed above). It is possible to evaluate such potential contributions, for example, by testing if the [buffer] and [salt] may be modified considerably without significant impact on the determined *K*_D(P)_ (i.e. if $\sum K_{A}[{\text{L}}_{\mathrm{ad}}]\ll 1$) [19]. However, unless contributions from all potential adventitious ligands can be ruled out, affinities should be confirmed by competition which is discussed next.

## Determination of metal–protein affinities via competition experiments

### Ligand-protein competition for metal

Consider a metal–protein complex whose affinity is beyond the tight limit of detection by direct metal titration (i.e. *K*_D(P)_ ≪ [P]): Under this condition, $\left([\text{MP}]/{\left[\text{M}\right]}_{\mathrm{tot}}\to 1\right)$ (equation 4) and the metal ions in the system are sequestered overwhelmingly by the protein with insignificant MP dissociation in solution. Such tight metal-binding prevents the formation of adventitious metal complexes, $\sum [\text{M}({\text{L}}_{\mathrm{ad}})]$, and reduces free metal ions, M_aq_, to a manageable concentration level (for example, Cu_aq_^+^ ions are thermodynamically stable at sub-nM concentrations [17]). However, in practice, it is impossible to quantify [M_aq_] directly at such low free metal concentrations. To determine a biologically meaningful conditional *K*_D(P)_ for a MP complex with *K*_D(P)_ ≪ [P], the complex may be made to partially dissociate to a reliably detectable level using a competing ligand, L, with a known conditional affinity, *K*_D(L)_, at a fixed pH as described by equation 7a:

(7a) $$\text{MP}+\text{L}⇌\text{ML}+\text{P }\;\;\;\;\;\;\;K_{\mathrm{ex}}=\;\frac{\left[\text{ML}][\text{P}\right]}{\left[\text{MP}][\text{L}\right]}=\frac{K_{D(P)}}{K_{D(L)}}$$

The ML complex may further react with the ligand L to produce a 1 : 2 complex, ML_2_. To simplify a system for determination of *K*_D(P)_, it is an advantage to choose those ligands and experimental conditions that favour formation of either a 1 : 1 or 1 : 2 complex, but not both, although the latter case can be handled [11]. The competition between a protein, P, and a 1 : 2 complex, ML_2_, is given by equation 7b:

(7b) $$\text{MP}+2\text{L }\;⇌\;M{\text{L}}_{2}+\text{P }\;\;\;\;\;\;\;\;\;\;\;\;K_{\mathrm{ex}}=\;\frac{\left[\text{M}{\text{L}}_{2}][\text{P}\right]}{[\text{MP}]{\left[\text{L}\right]}^{2}}=K_{D(P)\;}\beta_{2}\;$$

where *β*_2_ is the conditional accumulated formation constant of the ML_2_ complex at the specific pH of the experiment. Provided that one species of the metal exchange of equation 7a or 7b can be measured at equilibrium, the other components may be calculated via mass-balance. Then *K*_D(P)_ can be calculated relative to the known affinity of the competing ligand, *K*_D(L)_ or *β*_2_, via the exchange equilibrium constant, *K*_ex_. Thus, it is possible to determine the *K*_D(P)_ of a tightly bound MP complex at experimentally detectable protein concentrations (i.e. at [P] ≫ *K*_D(P)_) with reference to the known affinity of a competing ligand.

The conditional affinity of the competing ligand (i.e. *K*_D(L)_ or * β*_2_) may be determined directly at a given pH; or calculated at any pH from the known absolute values for the fully deprotonated ligands ($K_{D(L)}^{\mathrm{abs}}$ or $\beta_{2}^{\mathrm{abs}})$ and the p*K*_a_ of each donor atom using equation 7c:

(7c) $$K_{D(L)}=K_{D(L)}^{\mathrm{abs}}\;\alpha (\text{H});\;\beta_{2}=\beta_{2}^{\mathrm{abs}}\;[\alpha (\text{H}){]}^{2}$$

where $\alpha (\text{H})=(1+10^{\left(pK_{a1}-\mathrm{pH}\right)}+10^{\left((pK_{a1}+pK_{a2})-2\mathrm{pH}\right)}+\ldots )$. Absolute affinities and p*K*_a_ values for many ligands are available in ref. [18], and values for some commonly used ligands are listed in refs [10,11]. The conditional *K*_D(L)_ and *β*_2_ values at pH 7.0 for some commonly used ML and ML_2_-type probe ligands are given in Tables 3 and 4, respectively.

Usually, it is imperative to choose a competing ligand whose metal-binding properties are well-characterised since errors in the ligand affinity will be transmitted to the protein affinity. However, if the affinity of a ligand standard is unknown or controversial, a control experiment with a well-characterised competing ligand should be conducted in tandem under identical conditions as a calibration of both probe affinity and probe response. For example, the spectroscopic properties and formation constant *β*_2_ of chromophoric probe Zn^II^(Par)_2_ are highly pH-dependent with somewhat inconsistent literature values [27]. When it was used to determine the affinities of several Zn(II)-binding protein domains according to equation 7b, the classic metal ligand Egta, which is spectroscopically silent but has a well-characterised ‘absolute’ Zn(II) affinity (i.e. $K_{D(\mathrm{Egta})}^{\mathrm{abs}}$) that may be converted to conditional affinities for specific pH with known p*K*_a_ values [11], was used as an affinity calibrator [28,29]. Thus, neither the probe response nor the formation constant of Zn^II^(Par)_2_ had to be known accurately, since they both became relative values with the two similar tandem competitions for Zn(II) between probe ligand Par and protein P or control ligand Egta with a relationship $K_{D(P)}=\;K_{D(\mathrm{Egta})}K_{\mathrm{ex}(\mathrm{Egta})}/K_{\mathrm{ex}(P)}$ where *K*_ex_ is the respective exchange constant of equation 7b.

Critically, for any ligand competition experiments based on equation 7a or 7b, the relevant exchange equilibrium described by *K*_ex_ must be determined reliably. Thus, metal-binding to one of the competing components (P or L) must generate a quantifiable response which allows a reliable and sensitive quantification of the difference from the control response in the absence of the competing partner. This will ensure that small experimental errors in the measured response do not transmit to large errors in the derived *K*_D_. This can usually be achieved, provided:

- Metal-binding to one of the competing partners (P or L) is detected with high sensitivity (i.e. small relative error); and
- The response measured under the competitive condition is between 20% and 80% of the control response in the absence of the competition; and
- The starting concentrations of all competing partners are calibrated, and this is particularly important for the competing partner that is limiting in concentration.

Most commonly in ligand competition experiments, the metal, M, is the limiting reagent: In such cases the control response is dictated by the total metal concentration. Thus, the fraction of total metal-bound to P (and vice-versa to L) must be sensitively determined and should ideally be within the 0.2–0.8 range. The fraction of metal partitioned to the protein in the equilibria described by equations 7a and 7b may be expressed by equations 8a and 8b, respectively:

(8a) $$\frac{\left[\text{MP}\right]}{{\left[\text{M}\right]}_{\mathrm{tot}}}\;=\;\frac{1}{1\;+\;(K_{D(P)}/K_{D(L)})(\left[\text{L}]/[\text{P}\right])}$$

(8b) $$\frac{\left[\text{MP}\right]}{{\left[\text{M}\right]}_{\mathrm{tot}}}\;=\;\frac{1}{1\;+\;K_{D(P)\;}\beta_{2}({\left[\text{L}\right]}^{2}/[\text{P}])}$$

The apparent affinity, ^a^*K*_D(P)_, defined by equations 6a and 6b may apply broadly to equations 8a and 8b with ${}^{a}K_{D(P)}=K_{D(P)}([\text{L}]/K_{D(L)})$ and $K_{D(P)\;}({\left[L\right]}^{2}\beta_{2})$, respectively, but in this case the binding of the competing ligand, L, can be fully accounted for and the conditional *K*_D(P)_ can be accurately derived. Again, the most sensitive condition for the affinity determination is ^a^*K*_D(P)_ = [P], i.e. [MP]/[M]_tot_ = 0.5. This optimal condition may be achieved via experimental design by altering the relative concentrations of P and L in solution (see section ‘Design and optimisation of ligand or inter-metal competition experiments’) and/or by selecting a competing ligand with a well-matched affinity (see section ‘Spectroscopic probes as competing ligands and affinity standards’).

### Inter-metal competition for protein

By analogy to ligand competition of equation 7a, it is possible, in some cases, to set up a competition between two different metals, the readout metal ${\text{M}}^{R}\;$and the target metal ${\text{M}}^{T}$, for a single metal-binding site in a protein P, provided that the impact of adventitious binding to both free metals is negligible or can be suppressed (see section ‘Inter-metal competition experiments’ discussed below). In this case, protein P is limiting while both metals are in excess. The unknown affinity of ${\text{M}}^{T}$ for P (i.e. $K_{D(M^{T}P)}$) may be determined relative to the known affinity of ${\text{M}}^{R}$ (i.e. $K_{D(M^{R}P)}$) for the same (or equivalent) metal site in P via equation 9a:

(9a) $${\text{M}}^{R}\text{P }\;+{\;M}_{\mathrm{aq}}^{T}\;⇌\;{\text{M}}^{T}\text{P }\;+{\;M}_{\mathrm{aq}}^{R}\;\;\;\;\;\;\;\;\;K_{\mathrm{ex}}=\;\frac{\left[{\text{M}}^{T}\text{P}][{\text{M}}_{\mathrm{aq}}^{R}\;\right]}{\left[{\text{M}}^{R}\text{P}][{\text{M}}_{\mathrm{aq}}^{T}\right]}\;=\;\frac{K_{D(M^{R}P)}}{K_{D(M^{T}P)}}$$

Occupancy of the (limiting) protein with the target metal, M^T^, is not only dictated by the relative affinities of the two competing metals but also by their relative concentrations. These concentrations can be tuned to achieve an effective competition (i.e. 20–80% occupancy of each metal) according to equation 9b:

(9b) $$\frac{\left[{\text{M}}^{T}\text{P }\;\right]}{{\left[\text{P}\right]}_{\mathrm{tot}}}\;=\;\frac{1}{1\;+\;(K_{D(M^{T}P)}/K_{D(M^{R}P)})(\left[{\text{M}}_{\mathrm{aq}}^{R}\;]/[{\text{M}}_{\mathrm{aq}}^{T}\right])}$$

This strategy has proven especially useful for determining protein affinities of spectroscopically silent metal targets such as the closed d-shell metal ions Zn(II) and Cd(II) via reverse titration, a method pioneered initially by Berg et al. in their study of metal-binding properties of zinc finger peptides [30,31]. The protein-bound open d-shell metals Co(II) or Ni(II) usually exhibit characteristic charge transfer (CT) and d–d transitions in UV–visible or circular dichroism (CD) spectra and their replacement by Zn(II) may lead to loss of these spectroscopic features. Consequently, Zn(II)-binding to these protein sites may be evaluated according to equation 9a or 9b, either qualitatively or quantitatively, depending on the affinity difference of the chosen metal pair. For example, although the Co(II) affinities of several zinc finger peptides (at *K*_D_ ∼ 3 nM) are too tight to be determined by direct metal titration, they were determined readily by reverse Co^2+^ titration of the Ni(II)-peptide complex in the presence of excess Ni^2+^ in solution and the corresponding Zn(II) affinities were, in turn, determined via reverse Zn^2+^ titrations of the respective Co(II) complexes in the presence of excess Co^2+^ [23]. The determined affinities via such cascade reverse titrations matched those determined by ligand competition or potentiometry [23]. Similar examples have been reported for two zinc finger domains in poly(ADP-ribose) polymerase 1 protein [32]. A strategy to overcome the potential problem associated with adventitious attacks of the excess free metals is discussed later, in the section ‘‘Inter-metal competition experiments’.

## Design and optimisation of ligand or inter-metal competition experiments

A general principle for ligand competition experiments is to enable detection of a competitive metal-binding response that differs significantly (ideally within 20–80%) from the non-competitive response in at least some of the data set (e.g. in Figure 1B the most sensitive point of the titration is at [M]_tot_ = [P]_tot_). To this end, different strategies may be applied to experimental design and optimisation, depending on the nature of the detection probe and the availability of a suitable competing partner. In the next three sections, we consider the design of the most common type of ligand competition experiments where the metal, M, is the limiting reagent: In such cases, the binding of M to one of the two competing partners (P or L) must be sensitively detected and M must be measurably partitioned between P and L (defined as 20–80% distribution of M_tot_ between the two partners) to prevent small errors in detection of MP (or ML*_x_* where *x* = 1 or 2) being transmitted to large errors in derived *K*_D_ values. Firstly, the optimally matched competing partners may be selected based on the relative *K*_D(P)_, *K*_D(L)_ and *β*_2_ (discussed in section ‘Optimal conditions for both metal partitioning and metal occupancy’). Secondly, for the poorly matched competing partners, measurable metal partitioning may be achieved by optimising the relative concentrations of the two (discussed in section ‘Adjusting the application window of ligand probes by variation of [L]tot/[P]tot’) but potential impacts on detection sensitivity must be carefully assessed (discussed in section ‘Consequences of [P]tot/[L]tot variation and probe tolerance to low metal occupancy’). Then, we note that there is a special case where the protein (acting as the probe ligand) is limiting, i.e. [P]_tot _≪ [M]_tot_ < [L]_tot_, and the 20–80% metal partitioning rule does not apply but instead, a 20–80% probe occupancy rule supports reliable equilibrium measurements (discussed in section ‘Alternative experimental design where protein is the limiting reagent’). Finally, we consider the case of inter-metal competition with a strategy to avoid potential adventitious attacks of metal ions in excess (discussed in section ‘Inter-metal competition experiments’).

### Optimal conditions for both metal partitioning and metal occupancy

Consider a titration of metal, M, into an equimolar mixture of a protein, P, and competing ligand, L, assuming that both bind one equivalent of M competitively with high affinities, leaving negligible free M_aq_ in solution before both ligands are saturated (i.e. the situation described by equation 7a). Metal-binding responses detected by the probe (either P or L) can be simulated for different $\text{log}K_{\mathrm{ex}}=\text{log}\;(K_{D(P)}/K_{D(L)})$ and are shown in Figure 1B (here P is assumed to act as the detection probe). The binding isotherm for $\text{log}K_{\mathrm{ex}}=0$ symbolises equal affinities and is a diagonal straight line, indicating that the added metal ions are distributed evenly between the two competing partners. If $\text{log}K_{\mathrm{ex}}<0$, the probe P binds M with tighter affinity and the binding isotherms bend up but if $\text{log}K_{\mathrm{ex}}>0$, the competing ligand L binds M with tighter affinity and the isotherms bend down, indicating an unequal distribution of metal ions between the two competitors. A sensitive application window of the probe for $K_{D(P)}/K_{D(L)}$ may be approximately set to a probe response within the 20–80% range of the control response (in the absence of a competing partner) at the metal titration point of $[\text{M}{]}_{\mathrm{tot}}/[\text{P}{]}_{\mathrm{tot}}=1$, since the metal-binding isotherms are well-resolved within this range but converge rapidly outside this range towards one of the two non-competing metal-loading curves: Either that of the probe (the two oblique straight lines) or that of its non-probe partner (the two horizontal straight lines) (Figure 1B; black traces). This window covers a range of $1/16\leq K_{D(P)}/K_{D(L)}\;\leq 16/1$ according to equation 8a: Thus, the ideal application window of a metal probe P for reliable determination of *K*_D_ of itself or its competing partner is approximately $\text{log}K_{D(L)}=\text{log}K_{D(P)}\pm 1.2$ under the condition of [P]_tot_ = [L]_tot_ = [M]_tot_, and this optimal window remains unchanged when the concentration of each component changes proportionally. This scenario is described by case 3 of Table 1.

Similar considerations for the competition involving a 1 : 2 ML_2_ complex under the condition of [M]_tot_ = [P]_tot_ = ½ [L]_tot_ leads to an equivalent ‘optimal’ case 9 in Table 1 where log *K*_ex_ = 4.7 (i.e. $K_{D(P)}=10^{4.7}(\beta_{2}{)}^{-1}$) for [M]_tot_ = 10 µM or, more generally, $K_{D(P)}=(2{\left[\text{M}\right]}_{\mathrm{tot}\;}\beta_{2}{)}^{-1}$. This latter relationship between *K*_D(P)_ and $(\beta_{2}{)}^{-1}$ may be taken as an approximate comparison of the relative affinities between MP and ML_2_ complexes and it is noted that such comparison is [M]_tot_-dependent. For example, the $\beta_{2}$ values of four Cu(I) chromophoric probes Cu^I^L_2_ (L = Fs, Fz, Bca, Bcs; see Figure 4) have been determined to be 10^13.7^, 10^15.1^, 10^17.2^ and 10^19.8 ^M^−2^, respectively [8,33] and they are expected to have an ‘optimal’ application for protein targets with *K*_D(P)_ = 10^−9.5^, 10^−10.9^, 10^−13.0^ and 10^−15.6 ^M, respectively, when [Cu(I)]_tot_ = 30 µM. However, in practice, their optimal targeting *K*_D(P)_ values are about one order of magnitude tighter in affinity (see Figure 5) since these probe ligands are most reliably employed in excess (i.e. under condition [L]_tot_ > 2 [M]_tot_) to supress potential 1 : 1 complexes (see the arrows pointing to revised conditions of case 9 in Table 1). It is noted that *K*_D_ and$\;(\beta_{2}{)}^{-1}$, the metal affinities for 1 : 1 (ML) and 1 : 2 (ML_2_) complexes, have sometimes been compared directly but such comparison is incorrect and misleading, since these two constants, *K*_D_ and$\;(\beta_{2}{)}^{-1}$, have different units (M vs M^2^) and are neither equivalent nor directly comparable [11].

**Table 2.** Summary of the experimental conditions and determined affinities in Figure 3

Figure 3^1^	Probe ligand	Target	Exptl data	[Cu]_tot_ (µM)	[L]_tot_ (µM)	p[Cu^+^] at^2^		log*K*_D(P)_^3^
						Start	End	
b	Fs	CopK	Solid dots	31.0	90	9.1	10.3	<−9
			Empty dots	15.5	45	8.8	10.0	<−9
c	Fs	CopK	Solid dots	30.0	280	10.9	11.7	−11.2
			Empty dots	15.0	140	10.6	11.4	−11.2
d	Fz	CopK	Solid dots	30.0	120	11.2	11.8	−11.2
			Empty dots	15.0	60	10.9	11.9	−11.2
f	Bca	hGrx1	Solid dots	34.0	200	13.9	15.0	<−14
			Empty dots	34.0	100	12.7	14.5	<−13
g	Bca	hGrx1	Solid dots	38.0	500	14.9	15.4	−15.5
			Empty dots	19.0	250	14.6	15.2	−15.5
h	Bcs	hGrx1	Solid dots	32.0	80	14.7	15.8	−15.6
j	Bca	Atx1	Triangles	40	500	14.8	15.8	<−16
			Pluses	40	100	12.1	14.4	
		WNL5–6	Circles	40	500	14.8	15.8	<−16
			Crosses	40	100	12.1	14.3	
k	Bcs	Atx1	Solid dots	36	500	17.5	18.0	−17.7
			Empty dots		300	17.0	17.9	−17.7
l	Bcs	WLN5–6	Solid dots	36	500	17.5	18.0	−17.6
			Empty dots		200	16.4	17.6	−17.6

1 In Mops or KPi buffer (pH 7.0) containing excess reductant NH_2_OH and/or ascorbate;

2 Calculated based on the reported β_2_ Cu^I^L_2_ in refs. [8] and [33] (see Table 4);

3 Derived from fits of the experimental data to equation 14a (for expts. c,d,g,h,k,l) based on reported β_2_ for Cu^I^L_2_ given in refs. [8] and [33] (see Table 4).

**Table 3.** Probes that form 1 : 1 ML complexes with metal ions at pH 7.0

Probe	Metal	Detection	Signal	*K*_D_ (M)	Refs
Fura-2		Abs	*ε*_362 nm_ = 27 000 M^−1^ cm^−1^	* *	[61]
	Mn(II)	F (quenching)	*λ*_ex _∼ 340, *λ*_em _∼ 500 nm	2.8 × 10^−9^	[62]
	Co(II)	F (quenching)	*λ*_ex _∼ 340, *λ*_em_ ∼ 500 nm	8.6 × 10^−9^	[62]
	Fe(II)	F (quenching)	*λ*_ex _∼ 300–400 nm, *λ*_em _∼ 500 nm	n.d.^1^	[61]
	Ni(II)	F (quenching)	*λ*_ex _∼ 340, *λ*_em _∼ 500 nm	n.d.	[62]
	Zn(II)	F (change)	*λ*_ex _∼ 300–400 nm, *λ*_em _∼ 500 nm	2.0 × 10^−9^	[61]
Mf2		Abs	*ε*_369 nm_ = 29 900 M^−1^ cm^−1^		[66]
	Mn(II)	F (quenching)^2^	*λ*_ex _∼ 369 nm, *λ*_em _∼ 505 nm	9.7 × 10^−7 ^^3^	[67]
				6.1 × 10^−6 ^^3^	[5]
	Fe(II)	Abs^4^	Δ*ε*_366 nm_^5^ ∼ −20 000 M^−1^ cm^−1^	5.3 × 10^−6 ^^3^	[5]
	Co(II)	F (quenching)^2^	*λ*_ex_ ∼ 369 nm, *λ*_em_ ∼ 505 nm	9.3 × 10^−7 ^^3^	[67]
	Ni(II)	F (quenching)^2^	*λ*_ex_ ∼ 369 nm, *λ*_em_ ∼ 505 nm	1.3 × 10^−7^	[67]
			*λ*_ex_ ∼ 380 nm, *λ*_em_ ∼ 505 nm	3.3 × 10^−7^	[63]
			*λ*_ex_ ∼ 380 nm, *λ*_em_ ∼ 497 nm	5.0 × 10^−8^	[99]
	Zn(II)	F (change)^2^	*λ*_ex_ ∼ 330 nm, *λ*_em_ ∼ 505 nm	3.6 × 10^−8^	[67]
			*λ*_ex_ ∼ (320 and 345 nm)^6^, *λ*_em_ ∼ 505 nm	2.0 × 10^−8^	[100]
quin-2		Abs	*ε*_261 nm_ = 37 000 M^−1^ cm^−1^		[101]
	Zn(II)	Abs	Δ*ε*_265 nm_^5^ = 26 000 M^−1^ cm^−1^	3.7 × 10^−12^	[70]
DP1–4		Abs	*ε*_326 nm_ = 4500 M^−1^ cm^−1^		[19]
	Cu(II)	F (quenching)	*λ*_ex _∼ 330 nm, *λ*_em _∼ 550 nm		[19]
DP1	Cu(II)	F (quenching)	*F*_1_/*F*_0_ = 0.15 ^7^	7.9 × 10^−9 ^^8^	[19]
DP2	Cu(II)	F (quenching)	*F*_1_/*F*_0_*^ ^*= 0.17 ^7^	7.9 × 10^−11 ^^8^	[19]
DP3	Cu(II)	F (quenching)	*F*_1_/*F*_0_ = 0.13 ^7^	5.0 × 10^−13 ^^8^	[19]
DP4	Cu(II)	F (quenching)	*F*_1_/*F*_0_ = 0.09 ^7^	7.9 × 10^−15 ^^8^	[19]

1 Precise quantification prevented by experimental scatter, possibly a result of undefined Fe(II):fura-2 stoichiometry or imperfectly anaerobic conditions, see ref. [61];

2 Change in Mf2 absorbance (increase at ∼325 nm or decrease at ∼366 nm) can also be monitored; see refs [5,28,66];

3 Cautionary note: Mf2 probe may not be able to bind stoichiometric metal at equimolar metal concentrations in these cases (for ligands with *K*_D _> 10^−7 ^M, there will be >10% complex dissociation at [metal] = [protein] = 10 μM) and ‘free’ metal ions may be susceptible to attack from adventitious ligands (e.g. buffers) or metal hydrolysis. Particular care must be taken to ensure that metal speciation is accurately defined in these experiments;

4 Quenching of Mf2 fluorescence is also expected for paramagnetic (high spin) Fe(II);

5 Extinction coefficient corresponds to change in absorbance with respect to ligand only;

6 Ratio of fluorescence of Mf2 excited at 320 and 345 nm and emitted at 505 nm was used to quantify Zn(II) binding, see ref. [100];

7 Relative fluorescence (Cu(II)-probe/apo-probe);

8 At pH 7.4; properties are dependent on pH and the affinity data at pH 6.2 and 9.2 are also available, see ref. [19].

**Table 4.** Probe ligands that form 1 : 2 ML_2_ complexes with metal ions at pH 7.0

Ligand	Metal	Probe	*ε_λ_*_nm_ (M^−1 ^cm^−1^)	*β*_2_ (M^−2^)	Refs
Par^1^			*ε*_415 _∼ 37 800	* *	[78]
	Fe(II)	Fe^II^(Par)_2_	*ε*_705_ = 18 600	n.d.	[78]
	Co(II)	Co^II^(Par)_2_	Δ*ε*_514_ = 50 000 ^2^	n.d.	[79]
			Δ*ε*_508_ = 51 300 ^2^	n.d.	[27]
	Ni(II)	Ni^II^(Par)_2_	Δ*ε*_500_ = 52 000 ^2^	n.d.	[79]
	Zn(II)	Zn^II^(Par)_2_^3^	Δ*ε*_500_ = 66 000 ^2^	2.0 × 10^12^	[75]
			Δ*ε*_492_* *= 60 800 ^2^	4.7 × 10^11^	[27]
Tar^1^			*ε*_470_ = 24 800		[78]
	Fe(II)	Fe^II^(Tar)_2_	*ε*_720_ = 19 000 ^4^	4.0 × 10^13 ^^5^	[78]
	Fe(III)	Fe^III^(Tar)_2_	Δ*ε*_540_ = 46 500 ^2^	4.0 × 10^21 ^^6^	[78]
	Ni(II)	Ni^II^(Tar)_2_	Δ*ε*_535_ = 38 000 ^2^	4.3 × 10^15^	[6]
Fs^7^	Cu(I)	Cu^I^(Fs)_2_	*ε*_484_ = 6700	5.0 × 10^13^	[33]
Fz^7^	Cu(I)	Cu^I^(Fz)_2_	*ε*_470_ = 4320	1.3 × 10^15^	[33]
Bca^7^	Cu(I)	Cu^I^(Bca)_2_	*ε*_562_ = 7900	1.6 × 10^17 ^^8^	[8]
			Δ*ε*_358_ = 42 900 ^2^		[36]
				5.0 × 10^17 ^^8^	[24]
Bcs^7^	Cu(I)	Cu^I^(Bcs)_2_	*ε*_483_ = 13 000	6.3 × 10^19 ^^8^	[8]
				6.3 × 10^20 ^^8^	[24]

1 Spectral properties of Par and Tar are highly pH-dependent, see refs. [27,78,102];

2 Extinction coefficient corresponds to change in absorbance with respect to ligand only;

3 When working at micromolar-range concentrations, Par must be in excess to prevent dissociation of Zn(II) and formation of 1 : 1 Zn^II^Par complexes;

4 Value is pH-dependent and may be estimated by a relationship of *ε*_720_ = (4.14 pH–10) mM^−1 ^cm^−1^, see ref. [78];

5 Values at pH 7.2, 7.4 and 7.6 are also available, see ref. [78];

6 Calculated from the Nernst equation based on experimentally determined reduction potential (∼314 mV) of the Fe^III^(Tar)_2_/Fe^II^(Tar)_2_ redox couple [78];

7 Concentrations can be calibrated via titration of Cu(I) into respective ligand solutions as described in refs. [33,103];

8 Somewhat different values for Cu^I^(Bca)_2_ and Cu^I^(Bcs)_2_ have been reported, primarily due to a selection of different $E_{\mathrm{aq}}^{o}$ value for the aqueous Cu^2+^/Cu^+^ redox couple.

### Adjusting the application window of ligand probes by variation of [L]_tot_/[P]_tot_

The application windows discussed in the above section may be extended by lifting the restriction of [P]_tot_ = [L]_tot_ for ML or [P]_tot_ = ½ [L]_tot_ for ML_2_ to compensate for the difference in their relative affinities and thereby to maintain effective metal partitioning according to equation 8a or 8b. Table 1 describes a variety of experimental setups in which the condition $[\text{L}{]}_{\mathrm{tot}}/[\text{P}{]}_{\mathrm{tot}}$ may be optimised for different $\text{log}\;K_{\mathrm{ex}}$ to achieve an optimal metal distribution between P and L (i.e. $[\text{MP}]/[\text{M}{]}_{\mathrm{tot}}\;=0.5$). For example, consider a ligand L, with a 10-fold weaker affinity than the probe P (i.e. log *K*_ex_ = –1 in equation 7a). Under the condition of $[\text{L}{]}_{\mathrm{tot}}/[\text{P}{]}_{\mathrm{tot}}\;=1$, the probe detects a response that is 76% of the non-competitive control at [M]_tot_ = [P]_tot_ (the red trace in Figure 1B). However, upon increasing $[\text{L}{]}_{\mathrm{tot}}/[\text{P}{]}_{\mathrm{tot}}$ from 1 to 5.5, the probe response is shifted to its most sensitive position at 50% (Figure 1C). Indeed, even for a ligand L with a 100-fold weaker affinity (log*K*_ex_ = –2), the corresponding unreliable probe response of 91% at $[\text{L}{]}_{\mathrm{tot}}/[\text{P}{]}_{\mathrm{tot}}\;=1$ is shifted progressively to more reliable values of 79% and 50% of the non-competitive control when the $[\text{L}{]}_{\mathrm{tot}}/[\text{P}{]}_{\mathrm{tot}}$ ratio is increased to 5.5 and 50.5, respectively (Figure 1B–D, the green traces). Clearly, an increase in relative concentration of the competing ligand promotes MP complex dissociation. For competitions involving a 1 : 2 ML_2_ complex (equation 7b), calculations of metal partitioning via equation 8b also enable the design of a range of optimal experimental conditions that target different *K*_D(P)_, shown in cases 5–10 of Table 1.

### Consequences of [P]_tot_/[L]_tot_ variation and probe tolerance to low metal occupancy

Notably, the extension of the probe detection window to tighter or weaker affinity ranges comes at the expense of low metal occupancy on the weaker affinity partner, P or L, that must be present in excess to maintain the optimal 20–80% metal partitioning between the two (see Table 1). The consequence on the probe detection sensitivity must be carefully assessed. For example, consider a case of equation 7a where P acts as the detection probe and L is a ‘silent’ competitor, where both M and P are present in limiting concentrations and L is in excess (e.g. cases 1 and 2 in Table 1). This results in a low (<20%) metal occupancy on the L component but, provided that the silent ligand L does not interfere with the probe response and has negligible impact on [MP] detection, a low metal occupancy of a non-probe competitor is generally tolerated. In contrast, when M and L are limiting and the probe ligand P must be present in excess, the metal occupancy of the probe will fall below 20% (as in case 4): This, depending on the nature of the detection probe, may compromise the detection sensitivity. These different cases are analysed in detail next with various practical examples.

#### Turn-off probes and their restricted application windows

**Figure 2.**
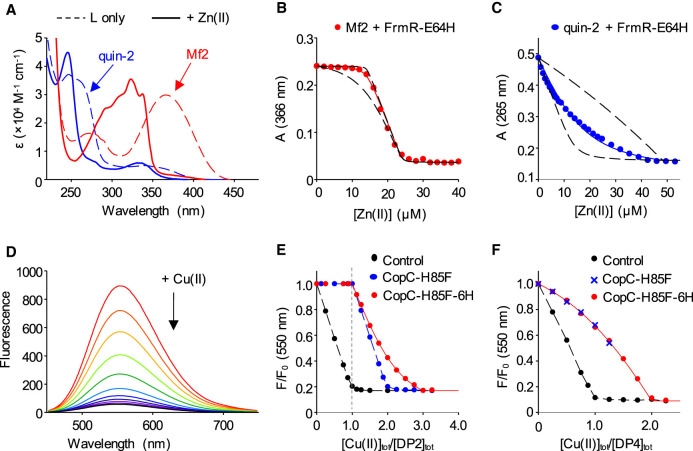
Examples of ‘turn-off’ probes (Mf2, quin-2 and DP peptides) and their restricted application windows. (**a**–**c**) Determination of Zn(II)-binding stoichiometry and affinity of protein FrmRE64H in 10 mM Hepes, pH 7.0, 100 mM NaCl, 400 mM KCl: (**a**) solution spectra of apo- and Zn(II)-bound probe ligands quin-2 (blue traces) and Mf2 (red traces); (**b**) Mf2 probe response upon titration of Zn(II) ions into a solution containing Mf2 probe (10.1 µM) and FrmRE64H (18.1 µM monomer); (**c**) quin-2 probe response upon titration of Zn(II) ions into a solution containing quin-2 probe (13.4 µM) and FmRE64H (42.7 µM). Solid lines are curve fits to a model describing protein competition with Mf2 or quin-2 for 0.75 equivalents of Zn(II) per FrmRE64H monomer (i.e. three sites per tetramer, *K*_Zn1–3_) generated using Dyanfit [60] (curves can also be modelled equivalently via equation 13b). Dashed lines are simulated curves with *K*_Zn1–3_ 10-fold tighter or weaker than the fitted values. Adapted from ref. [34] with data supplied by Dr. D. Osman and Prof. N.J. Robinson. (**d**–**f**) Determination of Cu(II) binding stoichiometry and affinity of protein CopC-H85F, with and without a hexa-His (6H) purification tag in 50 mM Mops pH 7.4: (**d**) quenching of fluorescence spectra of probe DP2 upon titration with Cu(II) ions; (**e**) DP2 probe response (plotted as normalised fluorescence, relative to apo-probe) upon titration of Cu(II) ions into a solution of DP2 only (4.0 µM, black); DP2 and CopC-H85F (4.0 µM each, blue); or DP2 and CopC-H85F-6H (4.0 µM each, red). (**f**) DP4 probe response upon titration of Cu(II) ions into a solution of DP4 only (4.0 µM, black); DP2 and CopC-H85F (4.0 µM each, blue); or DP2 and CopC-H85F-6H (4.0 µM each, red). Dashed black and blue lines in (**e**,**f**) are simple interpolation of data; solid red lines are curve fits via equation 13b to determine the weaker affinity Cu(II) site in CopC-H85F-6H (log *K*_D _= −9.4 in (**e**)) and the tight-affinity Cu(II) sites in CopC-H85F and CopC-H85F-6H (indistinguishable log *K*_D_ = −14.6 in (**f**)). Adapted from ref. [35].

If detection of metal-binding relies on a readout from the probe ligand itself, then it may be considered broadly to be a ‘turn-off’ probe. This type of probe detects its metal-loaded form *indirectly*, by reporting the relative concentrations of its metal-loaded and metal-free forms (i.e. probe response ∝[MP]/[P]_tot_). Consequently, if the probe must be present in excess with a low metal occupancy, its detection sensitivity is compromised. For this reason, a turn-off probe can generally only be applied to determine the metal affinity of a competitor with comparable or weaker affinity (i.e. a larger *K*_D_ value) but not to the one with stronger affinity, and its application window is restricted by the condition: $\text{log }\;K_{D(L)}\geq \text{log}\;K_{D(P)}-1.2$. For example, a metal-responsive variant of the *Salmonella typhimurium* formaldehyde sensor (FrmRE64H), which binds four Zn(II) ions per protein tetramer with varying affinities, competed for Zn(II) overwhelmingly against probe mag-fura-2 (Mf2; *K*_D(L)_ ∼ 20 nM at pH 7.0) for the tightest three sites at a level of <10% probe response (Figure 2A,B). This experiment, with only weak metal competition from the probe, allowed a quick estimation of a (weak) affinity limit for these three metal sites at the sub-nM level [34]. However, the excess concentration of Mf2 that would be required to impose an *effective* competition with FrmRE64H for Zn(II) (e.g. case 1 in Table 1) could not be employed while retaining the necessary detection sensitivity. Thus, a subsequent competitive experiment with a better-matched probe of higher affinity, quin-2 (*K*_D(L)_ = 3.7 pM at pH 7.0) was employed to provide a more reliable estimation of the average Zn(II) affinity of the three tight sites (*K*_D(P)_ = 23 pM at pH 7.0) (Figure 2C) [34]. Similarly, a variant of the high-affinity Cu(II)-binding protein CopC from *Pseudomonas fluorescens* (CopC-H85F) withheld one equivalent of Cu(II) completely from the turn-off fluorescent probe DP2 (*K*_D(L)_ = 790 pM at pH 7.4) but competed effectively with probe DP4 of femtomolar (fM) affinity (*K*_D(L)_ = 7.9 fM at pH 7.4), enabling a protein affinity of *K*_D(P)_ = 2.5 fM to be determined reliably (Figure 2D–F) [35]. Interestingly, the presence of a hexa-His tag (in CopC-H85F-6H) generated an additional, weaker Cu(II) site ($K_{D(\mathrm{His}-\mathrm{tag})}\;=400\;\text{pM}$ at pH 7.4) that could be quantified reliably with probe DP2 of comparable affinity (Figure 2E) but had no detectable impact on quantification of the fM affinity site in CopC by the probe DP4 (Figure 2F; note the indistinguishable Cu(II) competitions between DP4 and CopC-H85F or CopC-H85F-6H) [35]. This example also indicates that a purification tag will interfere with the characterisation of native site(s) of comparable affinity but not with native site(s) of much tighter affinity.

#### Turn-on probes and their flexible application windows

**Figure 3.**
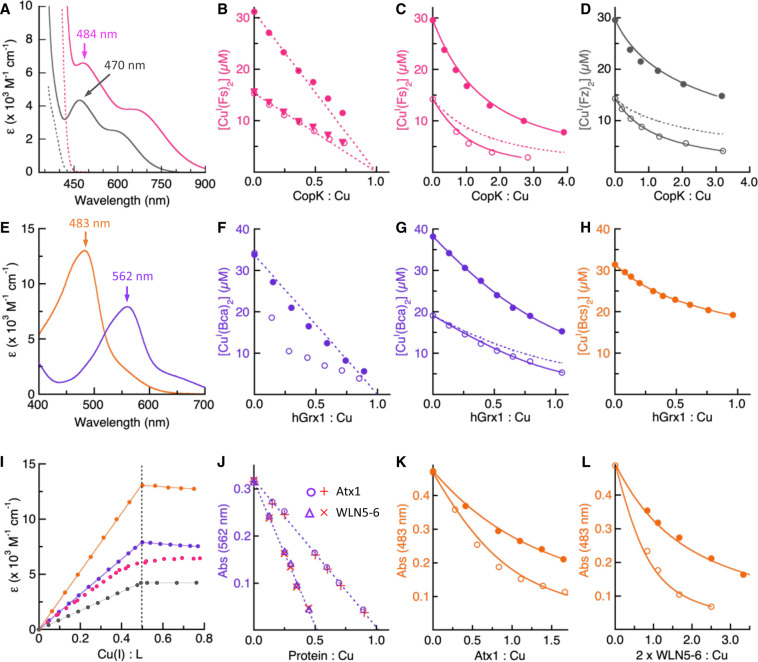
Examples of the flexible application of ‘turn-on’ chromophoric probes Cu^I^L_2_ (L = Fs, Fz, Bca, Bcs). (**a**) Solution spectra of Cu^I^(Fs)_2_ (magenta) and Cu^I^(Fz)_2_ (grey) and the respective apo-probe ligands (dashed traces). (**b**) Determination of Cu(I)-binding stoichiometry of CopK protein with Cu^I^(Fs)_2_. Change in probe concentrations (monitored by absorbance) with increasing [CopK] in a series of assay solutions (details in Table 2). The data points in triangles show the 50% values of the solid circles, demonstrating that the equilibrium position in each diluted solution has not noticeably adjusted from that present in the corresponding undiluted solution (consistent with stoichiometric binding). It was apparent that CopK could bind one equivalent of Cu(I) with sub-nM affinity. (**c**,**d**) Determination of Cu(I)-binding affinity of CopK with Cu^I^(Fs)_2_ (**c**) or Cu^I^(Fz)_2_ (**d**) (details in Table 2). The solid traces are data fits to equation 14a, deriving *K*_D(P)_ given in Table 2. The two dashed traces shows the 50% values of the filled circles and demonstrate that the equilibrium position in each diluted solution has adjusted from that present in the corresponding undiluted solution. (**e**) Solution spectra of Cu^I^(Bca)_2_ (purple) and Cu^I^(Bcs)_2_ (orange) (neither probe ligand has absorbance in the given visible window). (**f**) Determination of Cu(I)-binding stoichiometry of hGrx1 protein with the Cu^I^(Bca)_2_ probe (details in Table 2). It was apparent that hGrx1 could bind more than one Cu(I) with *K*_D(P) _< 10^−13^ M but only one Cu(I) with *K*_D(P) _< 10^−14^ M at pH 7.0. (**g**,**h**) Determination of Cu(I)-binding affinity of hGrx1 with Cu^I^(Bca)_2_ (**g**) or Cu^I^(Bcs)_2_ (**h**) (details in Table 2). The solid traces are the data fits to equation 14a, deriving *K*_D(P)_ given in Table 2. The dashed trace in (**g**) shows the 50% values of the filled circles and demonstrates that the equilibrium position in each diluted solution has adjusted from that present in the corresponding undiluted solution. (**i**) Cu(I) titration of each probe ligand under reducing conditions followed by absorbance at the λ_max_ (nm) given in (**a**,**e**): a tight turning point was observed for ligands Bcs, Bca and Fz, but not for Fs due to its relative weak affinity for Cu(I). Thus, presence of an excess of Fs is essential for its application. (**j**,**k**,**l**) Quantification of Cu(I)-binding to yeast Atx1 and human WLN5–6 in KPi buffer (pH 7.0): (**j**) determination of respective Cu(I)-binding stoichiometry with two assay solutions of Cu^I^(Bca)_2_ described in Table 2; determination of aM affinity of Atx1 (**k**) and WLN5–6 (l) with two different assay solutions of Cu^I^(Bcs)_2_ described in Table 2. The solid traces are the data fits to equation 14a, deriving *K*_D(P)_ given in Table 2. Data (**a**–**d**, **i**), (**f**–**h**) and (**j**–**l**) adapted from refs. [33], [37] and [8], respectively.

If detection of metal-binding to a probe ligand relies on a readout from its metal complex, that is independent of the probe ligand itself, it may be considered broadly to be a ‘turn-on’ probe. A turn-on probe detects its metal-loaded form *directly* with no restriction on the concentration of its metal-free form. Therefore, a low metal occupancy is generally well-tolerated and does not affect the detection sensitivity. For this reason, a turn-on probe is more flexible and versatile in its application than a turn-off probe. This is demonstrated nicely by the applications of the above-mentioned four Cu(I) probes Cu^I^L_2_ (L = Fs, Fz, Bca, Bcs) for quantification of the Cu(I)-binding properties of various protein targets. These probes are chromophoric ML_2_-type Cu(I) complexes with characteristic absorbance in the visible spectral range. They display different detection sensitivity but the probe ligands themselves have little absorbance above 450 nm (Figure 3A,E,I), and thus they may be classified as turn-on Cu(I) probes. The difference in their formation constants mean that their optimal targeting affinities are different (see section ‘Optimal conditions for both metal partitioning and metal occupancy’ discussed above), but by varying the silent probe ligand concentrations, their *effective* application windows may be significantly extended to cover a wide spectrum of affinities under different experimental conditions as suggested for the various cases in Tables 1 and 2. For example, the weakest probe Cu^I^(Fs)_2_ has a predicted ‘best-matched’ targeting affinity of *K*_D(P)_ ∼ 10^−9.5 ^M at [M]_tot_ = 30 µM (section ‘Optimal conditions for both metal partitioning and metal occupancy’) and was proved to be an ideal probe for quantification of the Cu(I)-binding stoichiometry, under a non-competitive condition (close to case 8 in Table 1), of a bacterial copper-binding protein CopK, but with an increase in probe ligand Fs concentration (close to case 7 in Table 1), the same probe determined a reliable *K*_D(P)_ = 10^−11.2 ^M at pH 7.0 for CopK (see Figure 3B,C and Table 2) [33]. This *K*_D(P)_ value was confirmed independently by the better-matched probe Cu^I^(Fz)_2_ (whose ‘optimal’ targeting affinity was predicted to be *K*_D(P)_ = 10^−10.9 ^M at [M]_tot_ = 30 µM; with experimental conditions close to case 8 (Figure 3D; Table 2). Even the probe Cu^I^(Bca)_2_, which has a much tighter predicted ‘optimal’ targeting affinity (*K*_D(P)_ ∼ 10^−13.0 ^M), was employed to determine the CopK affinity under an extreme condition, close to case 10, with a large excess of CopK (100–400 µM) but limiting Bca (45 µM) [36]. However, the estimated *K*_D(P)_ = 10^−10.7^ M was modestly larger (weaker in affinity) than the *K*_D(P)_ = 10^−11.2^ M determined with the Cu^I^(Fs)_2_ or Cu^I^(Fz)_2_ probes. The difference may have arisen from two sources: (i) apo-CopK exists in a monomer-dimer equilibrium (*K*_D_ ∼ 0–50 µM) and was present mainly as an apo-dimer at high concentrations (>100 µM) required with the Cu^I^(Bca)_2_ assay but dissociated to an apo-monomer at lower concentrations (<70 µM) required with the Cu^I^(Fs)_2_ or Cu^I^(Fz)_2_ assays. However, Cu(I)-binding dissociated apo-CopK dimer into Cu^I^-CopK monomer at all concentrations and thus the Cu(I)-binding equilibria at high and low CopK concentrations were different; (ii) for any assay involving two competing ligands with large differences in concentrations and thus in metal occupancies, the experimental errors, although less sensitive to the concentration of the component with lower metal occupancy (CopK in this case), become much more sensitive to the concentration of the component with higher metal occupancy (the ligand Bca in this case): A small error in Bca concentration may transmit larger error to the derivation of *K*_D(P)_. For this second reason, experimental conditions that may lead to very low metal occupancy on a competing partner, such as cases 5 and 10 in Table 1, should be avoided where possible by choosing a ‘better-matched’ competing partner for a given protein target (see section ‘Optimal conditions for both metal partitioning and metal occupancy’).

**Figure 4.**
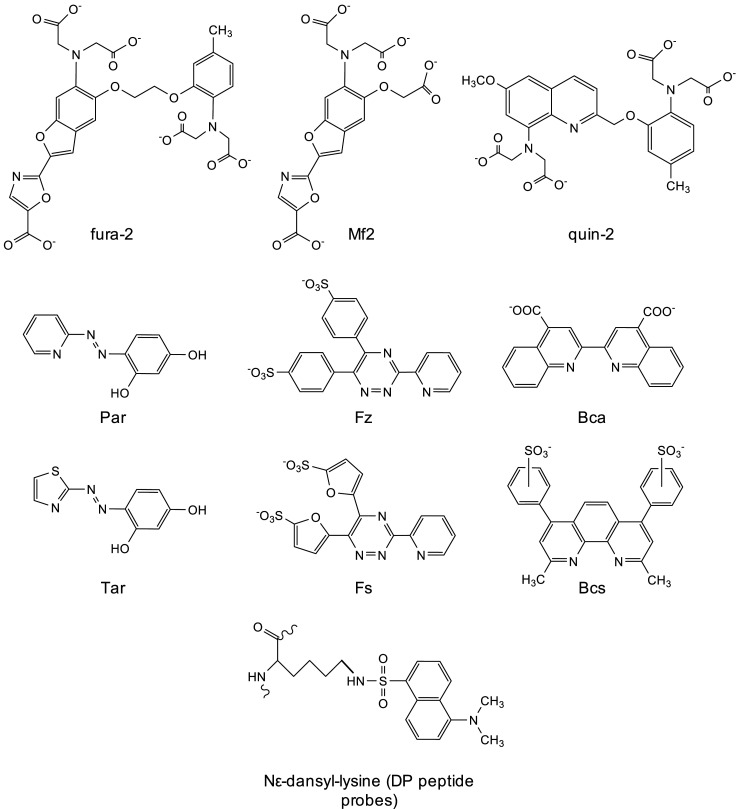
Structure of selected metal probe ligands. Structure of ML-type (fura-2, Mf2, quin-2) and ML_2_-type (Par, Tar, Fs, Fz, Bca and Bcs) probe ligands, and of the fluorescent N*ε*-dansyl-lysine that is incorporated into each DP peptide probe (see ref. [19] for complete peptide structures).

**Figure 5.**
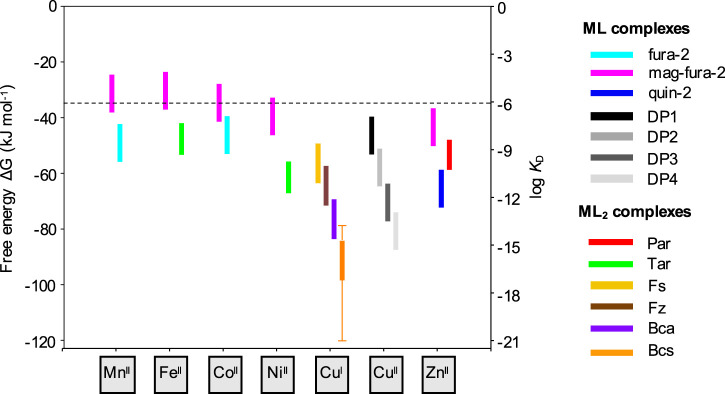
Approximate application ranges of selected spectroscopic probes for affinity determination. Ranges of metal–protein affinities (as dissociation constants, *K*_D_, and as free energies for metal association, Δ*G*, where Δ*G* = *RT* ln*K*_D_) that may be reliably determined via competition with spectroscopic probes using typical experimental setups: For ML-type probes (fura-2, mag-fura-2, quin-2, DP1–4), ranges correspond to optimal 20–80% metal partitioning in a solution containing equimolar metal, protein and probe (e.g. 10 µM each. For Tar, range corresponds to 20–80% metal partitioning in a solution of metal (10 µM), protein (10 µM) and Tar (20 µM). For Par, range corresponds to 20–80% metal partitioning in a solution of metal (10 µM), protein (10 µM) and Par (100 µM) (excess [Par] supresses 1 : 1 complex formation; see section ‘Par’). For Fs, Fz, Bca and Bcs range corresponds to 20–80% metal partitioning in a solution of metal (30 µM), protein (30 µM) and probe (75 µM) (increasing [Cu(I)] to 30 µM enhances detection sensitivity of these probes, see Table 4). However, in practice, the application windows may be extended beyond the presented ranges by altering the experimental design (see section ‘Design and optimisation of ligand or inter-metal competition experiments’): an example is given for probe Bcs where extended bars show measurable affinity ranges for experimental setups with excess protein (200 µM, weaker limit) or excess Bcs (5 mM, tighter limit). All calculations are based on metal–probe affinities determined at pH 7.0, except those for probes DP1–4 which were determined at pH 7.4 (see Tables 3,4). The dashed line indicates that care should be taken to ensure metal speciation remains well defined where weaker probes are employed (see section ‘Non-competitive controls, medium pH and buffers’ and footnotes in Table 3); noting that weak metal–protein affinities (*K*_D _> 10^−7 ^M) may be quantified using direct metal titration method with a careful control of the solution conditions (see section ‘Determination of metal–protein affinities via direct metal titration’).

The Cu^I^(Bcs)_2_ probe has a predicted optimal sub-fM targeting affinity and is far too strong to allow a meaningful evaluation of the Cu(I) affinity of CopK that is weaker by over four orders of magnitude. However, Cu^I^(Bcs)_2_ was an ideal probe for human glutaredoxin protein (hGrx1) and determined a Cu(I) *K*_D(P)_ = 10^−15.6 ^M at pH 7.0 with conditions close to the revised case 9 in Table 1 (see Figure 3H and Table 2) [37]. This value matched *K*_D(P)_ = 10^−15.5 ^M determined with the weaker probe Cu^I^(Bca)_2_ in the presence of a large excess of probe ligand Bca, at conditions between case 6 and case 7 in Table 1 (see Figure 3G and Table 2) [37]. The Cu^I^(Bca)_2_ probe is too weak to allow a quantitative evaluation of the attomolar (aM) Cu(I) affinities of yeast Cu(I)-carrying protein Atox1 or the linked N-terminal metal-binding domains 5 and 6 of human Wilson disease protein, WLN5–6, even in the presence of a large excess of Bca ligand, a condition similar to case 5 in Table 1. However, this does mean that Cu^I^(Bca)_2_ is an excellent probe to quantify the Cu(I) stoichiometries of both Atx1 and WLN5–6 (see Figure 3J) [8]. Notably, the determined Cu(I) stoichiometries for Atx1 (*n* = 1) and WLN5–6 (*n* = 2) remained unchanged with free [Cu^+^] varying between pCu^+^ = 12–16 (see the entry j in Table 2), suggesting that there were no other competing Cu(I) sites within this affinity window (this is in contrast to the case of hGrx1 in Figure 3F). The aM affinities of both proteins were confirmed consistently with the Cu^I^(Bcs)_2_ probe using two separate assay conditions close to cases 5 and 6 in Table 1 (see Figure 3K,L and Table 2) [8]. It is also noted that, in the presence of 5 mM Bcs ligand, a condition beyond case 5 of Table 1, the Cu^I^(Bcs)_2_ probe was used to quantify the extreme zeptomolar (zM) Cu(I) affinities of two high-affinity Cu(I)-binding methanobactin peptides from *Methylosinus trichosporium* OB3b [38]. It is concluded from these demonstrated examples that: (i) the application windows of the ML_2_-type turn-on probes may be extended from their predicted optimal targeting affinity considerably, as suggested from the modelling cases 5–10 of Table 1; (ii) the experiments modelled in Table 1 may be executed via different experimental procedures (by variation of [M]_tot_, [P]_tot_ or [L]_tot_) but with consistent outcomes.

### Alternative experimental design where protein is the limiting reagent

Ligand competition experiments may be designed such that the protein is the limiting reagent in a solution of ligand-buffered metal (i.e. [P]_tot_ ≪ [M]_tot_ < [L]_tot_). In such cases the protein itself must act as a sensitive detection probe. Since the control response of the experiment is dictated by the total protein concentration, the experimental data must cover metal occupancies of the protein ([MP]/[P]_tot_) within the 20–80% range to enable *K*_D(P)_ to be determined reliably (see section ‘Determination of metal–protein affinities via competition experiments’). The fractional metal occupancy of the protein is dictated by the concentration of free metal, [M_aq_], in solution as described by equation 10 which, in turn, varies with the concentrations of metal-bound and metal-free ligand in solution as described by equation 11a (for ML complexes) or equation 11b (for ML_2_ complexes):

(10) $$\frac{\left[\text{MP}\right]}{{\left[\text{P}\right]}_{\mathrm{tot}}}\;=\;\frac{1}{1\;+\;(K_{D(P)}/[{\text{M}}_{\mathrm{aq}}])}$$

(11a) $$\frac{\left[\text{MP}\right]}{{\left[\text{P}\right]}_{\mathrm{tot}}}\;=\;\frac{1}{1\;+\;(K_{D(P)}/K_{D(L)})(\left[\text{L}]/[\text{ML}\right])}$$

(11b) $$\frac{\left[\text{MP}\right]}{{\left[\text{P}\right]}_{\mathrm{tot}}}\;=\;\frac{1}{1\;+\;(K_{D(P)}\beta_{2})({\left[\text{L}\right]}^{2}/[\text{M}{\text{L}}_{2}])}\;$$

It is apparent from equations 11a and 11b that an optimal protein metalation of [MP]/[P]_tot_ = 0.5 may be achieved by adjusting the concentrations of L and ML*_x_* (*x* = 1 or 2) to compensate for differences in the relative affinities of P and L. Here, the couple [ML*_x_*]/[L] acts effectively as a metal buffer controlling metal availability to the protein target.

One illustrative example of this type of experimental design is the determination of the Cu(II) affinity of labile sites in the multicopper oxidase, CueO, using Bis-Tris pH buffer as the competing ligand [20]. The phenol oxidation activity of the enzyme relies on Cu(II) occupation of the labile sites [39] and was used as a sensitive turn-on probe to quantify the Cu(II)-binding to these sites. However, their average Cu(II) affinity (*K*_D _∼ 5 nM) was over 1000 times stronger than that of Bis-Tris at pH 7 (*K*_D_ ∼ 7 µM) and required a large excess (mM level) of Bis-Tris to impose an effective competition [20]. By supplementing 50 mM Bis-Tris with different concentrations of [Cu(II)]_tot_ (10–500 µM), a series of Cu^2+^-buffers with different but stable and well defined [Cu_aq_^2+^] (ranging from 1 to 30 nM) were produced. This enabled the fractional metalation of the labile sites to be varied according to equation 10 and the CueO enzyme (present at only 0.1 µM) was found to exhibit a 50% maximal activity at [Cu_aq_^2+^] ∼ 5.5 nM, thus an average Cu(II) affinity of these labile sites was determined [20].

Other similar examples where protein was used as the limiting reagent in the presence of excess ligand-buffered metal include the quantification of Zn(II)-binding to variant forms of the enzyme carbonic anhydrase by competition with dipicolinic acid (DPA) [40], quantification of Zn(II)-binding to the metalloregulator ZntR by competition with TPEN [41] and quantification of Cu(I)-binding to the fluorescent probe CS1 by competition with thiourea (where CS1 acts as ‘P’) [42]. In each case the concentration of metal-bound protein was sensitively detected, with a metal occupancy covering the range between 20% and 80% at numerous points in the titration, enabling reliable measurements of the competition equilibrium *K*_ex_ and thus *K*_D(P)_.

### Inter-metal competition experiments

For the inter-metal competition of equation 9a, the two competing metal ions are assumed to be present as hydrated free forms (usually in the µM–mM range). This assumption could be a problem in certain cases due to the possible attack of these ‘free’ metals by adventitious ligands (see section ‘Adventitious metal ligands and the apparent dissociation constant’). Consequently, the term $\left[{\text{M}}^{R}{}_{\mathrm{aq}}]/[{\text{M}}_{\mathrm{aq}}^{T}\right]$ in equation 9a may need to be modified by a factor of $\left(1+\;\sum K_{A}^{R}[{\text{L}}_{\mathrm{ad}}^{R}]\right)/\left(1+\;\sum K_{A}^{T}[{\text{L}}_{\mathrm{ad}}^{T}]\right)$. This problem is generally partially offset by the ratio relationship between $\left(1+\;\sum K_{A}^{R}[{\text{L}}_{\mathrm{ad}}^{R}]\right)$ and $\left(1+\;\sum K_{A}^{T}[{\text{L}}_{\mathrm{ad}}^{T}]\right)$ but may not be eliminated completely due to the likely unequal values of the $\sum K_{A}[{\text{L}}_{\mathrm{ad}}]$ terms for different metals. This problem, if substantial, may be alleviated by introducing into the system a relatively weak protecting ligand L in excess (i.e. $[\text{L}{]}_{\mathrm{tot}}>[{\text{M}}^{R}{]}_{\mathrm{tot}}+[{\text{M}}^{T}{]}_{\mathrm{tot}}$) on the condition that the ligand L should have little competition with protein P for either metal, M^R^ or M^T^, but must have sufficient affinities to protect all protein-unbound metals in accountable complexes, ${\text{M}}^{R}\text{L}$ and ${\text{M}}^{T}\text{L}$. Then equation 9a may be modified to equation 12a with conditions of equations 12b–12e:

(12a) $${\text{M}}^{R}\text{P }\;+\;{\text{M}}^{T}\text{L }\;⇌\;{\text{M}}^{T}\text{P }\;+\;{\text{M}}^{R}\text{L }\;\;\;\;K_{\mathrm{ex}}=\;\frac{\left[{\text{M}}^{T}\text{P}][{\text{M}}^{R}\text{L}\;\right]}{\left[{\text{M}}^{R}\text{P}][{\text{M}}^{T}\text{L}\right]}\;=\;\frac{K_{D(M^{R}P)}}{K_{D(M^{T}P)}}\times \frac{K_{D(M^{T}L)}}{K_{D(M^{R}L)}}$$

(12b) $$\left[{\text{M}}^{R}{]}_{\mathrm{tot}}=\;[{\text{M}}^{R}\text{P}]\;+\;[{\text{M}}^{R}\text{L}\right]$$

(12c) $$\left[{\text{M}}^{T}{]}_{\mathrm{tot}}=\;[{\text{M}}^{T}\text{P}]\;+\;[{\text{M}}^{T}\text{L}\right]$$

(12d) $$\left[\text{P}{]}_{\mathrm{tot}}=\;[{\text{M}}^{R}\text{P}]\;+\;[{\text{M}}^{T}\text{P}\right]$$

(12e) $$[\text{L}{]}_{\mathrm{tot}}>\;[{\text{M}}^{R}{]}_{\mathrm{tot}}+\;[{\text{M}}^{T}{]}_{\mathrm{tot}}$$

The first two conditions (equations 12b and 12c) are satisfied provided that: (i) the conditional affinities of the ligand L for each metal at the experimental pH are tight enough to sequester all protein-unbound metal ions; (ii) equation 12e is satisfied. The third condition (equation 12d) is determined by the relative metal affinities of the protein P and the ligand L and the relative concentrations of the free and metalated ligand. Finally, of course, the probe metal complexes ${\text{M}}^{R}\text{P}$ and ${\text{M}}^{R}\text{L}$ must have distinct spectroscopic readouts to ensure an accurate experimental evaluation of the equilibrium 12a.

This approach was pioneered very recently by an example that determined the Zn(II) affinity of a Co(II)-carrying protein CobW that helps to deliver and insert an essential Co(II) ion into vitamin B_12_ [6]. This protein, upon binding nucleotide (GTP) and Mg(II), assembles a metal site that can bind either Co(II) or Zn(II) with high affinity [6]. The Co(II)-bound form exhibits characteristic S^–^ → Co(II) ligand to metal charge transfer (LMCT) absorbance in the near UV region, providing a convenient direct probe for the affinity determination by classic ligand competition (*K*_D(Co)_ = 10^−10.5 ^M at pH 7.0). The affinity for the spectroscopically silent Zn(II) (*K*_D(Zn)_ = 10^−12.7 ^M at pH 7.0) was determined subsequently by reverse Zn^2+^ titration into the pre-formed Co^II^-CobW-Mg^II^GTP complex in the presence of various concentrations of excess Co^2+^ ions that were fully protected by ligand nitrilotriacetic acid (NTA; *K*_D(Co-NTA)_ = 10^−7.6 ^M and *K*_D(Zn-NTA)_ = 10^−7.9 ^M at pH 7.0) [6]. Here, the NTA affinities for both metals were weaker than the corresponding protein affinities by three (or more) orders of magnitude, ensuring little competition (with the protein) from NTA for either metal. On the other hand, the NTA affinities for both metals are high enough to provide adequate protection for all protein-unbound metal ions.

Notably, the NTA affinities for Co(II) and Zn(II) are very close, demonstrating the strong offsetting effect of the NTA chelation on equation 9a. The Zn(II) affinity for a protein determined by the reverse titration method depends heavily on the initially determined Co(II) affinity for the same protein site and therefore, a primary source of discrepancy in Zn(II) affinity determinations is more likely due to an unreliable reference Co(II) affinity and less likely to the technique of the reverse titration itself. Nevertheless, the competition based on equations 12(a-e) will ensure an elimination of the method error due to adventitious bindings. In addition, in this example the protein affinities for both metals, although differing by about two orders of magnitude, are still within the manageable range to allow optimisation of the experimental conditions to establish an effective competition for a reliable determination (see discussion in section ‘Design and optimisation of ligand or inter-metal competition experiments’) [6]. This example may provide a useful guide for a systematic re-evaluation of Zn(II) affinities to various zinc finger peptides via the reverse titration and NTA may be an excellent protecting ligand for many applications. But, of course, the reference affinities of the probe metals, Co(II) and Ni(II), to the proteins or peptides must be determined reliably first (see section ‘Illustrative examples of metal affinity determination by direct metal titration’ and refs. [9,23]).

## Readouts of metal-binding equilibria

Any biophysical or biochemical response or process capable of quantifying the concentration of any single species of equations 3, 7a or 7b at equilibrium may be employed for *K*_D(P)_ determination [11,43]. Common readouts for detection and analysis may be catalogued into three broad classes: (i) direct analysis of protein metal occupancy via equilibrium separation, followed by direct metal quantification; (ii) analysis of the reaction enthalpy generated from metal-binding to the target protein via isothermal titration calorimetry (ITC); and (iii) direct detection and analysis of one or more species in the metal-binding equilibrium via spectroscopic methods.

### Direct metal analysis

This method reported the first attempt in quantification of metal-binding in a protein [44]. It requires separation of protein components from the equilibrated metal-containing buffer without perturbing the established metal-binding equilibria, followed by direct metal analysis of the separated fractions. Depending on the metal affinity of the protein target, the metal availability (i.e. p[M] = −log [M]) of the metal buffer may need to be adjusted by various metal-buffer ligands such as Tris, Bis-Tris, glycine, histidine, or even Egta and Edta, etc., to ensure a fractional metal occupation on protein within the 0.2–0.8 range for a sensitive and reliable analysis (see section ‘Design and optimisation of ligand or inter-metal competition experiments’). The separation may be accomplished by a protein-impermeable membrane such as a diaflow filtration unit or a dialysis chamber [40,45], by protein sedimentation via ultracentrifugation [46] or by chromatographic separation via a column elution [47,48]. The metal analysis is nowadays undertaken routinely by robust approaches such as atomic absorption spectroscopy (AAS) and inductively coupled plasma mass spectrometry (ICP-MS). These methods offer an advantage that almost any metal ion can be analysed. However, with the exception of chromatography, these methods generally require very extensive equilibrium time (hours to days) and may not work for small proteins or peptides; while the chromatographic separation may cause some disturbance to the established binding equilibria, especially for those proteins with labile metal-binding kinetics and thus may not be suitable for quantitative analysis in such cases.

This method is normally conducted under the condition [P]_tot _∼ [M]_tot_ for weak binding or [P]_tot_ ≤ [M]_tot_ < [L]_tot_ for tight binding and so the principle and equations 10, 11a, 11b discussed in the above section ‘Alternative experimental design where protein is the limiting reagent’ apply. For dialysis separation, the metal content of the protein, [MP], at equilibrium is the difference between the metal concentrations of the two separated fractions and so the metal occupancy on the protein, [MP]/[P]_tot_, may be obtained if the [P]_tot_ of the protein-containing fraction is determined. For diaflow filtration or ultracentrifugation separation, the [MP] at equilibrium is the difference between the metal concentrations of the protein solution before the filtration (or centrifugation) and the protein-free solution after the separation. The protein concentration before the separation is taken as [P]_tot_ for the metal occupancy calculation. Notably, the total protein concentration in the filtration or centrifugation process changes constantly during the course of the separation but this does not change the initial metal-binding equilibria established before the separation, provided that (i) both metal-free and metal-containing protein species contain the same protein stoichiometry (this indeed is the case for equations 3, 7a and 7b); (ii) all non-protein components can pass the membrane freely or cannot be sedimented while all protein components are impermeable to the membrane or sedimented completely off the top solution to be taken for the metal analysis.

### ITC analysis

The ITC method quantifies the reaction enthalpy (Δ*H^o^*) of metal-binding to a protein and thus offers an obvious advantage that the method may be applicable to any metal-binding event and can derive a set of thermodynamic parameters including reaction enthalpy (Δ*H^o^*), entropy (Δ*S^o^*) and binding affinity (*K*_D_). However, this also means that the method lacks specificity: All coupled reactions (such as dilution, buffer or specific ligand competition and proton displacement) or unwanted side reactions (such as metal hydrolysis and redox) could make substantial contributions to the experimental enthalpy *ΔH*_ITC_ which could be very different from the targeting Δ*H^o^* specific to equation 3 that is required for an accurate derivation of *K*_D(P)_. Consequently, great care must be taken in experimental design and execution to avoid unwanted side reactions and to ensure effective binding competition (see section ‘Design and optimisation of ligand or inter-metal competition experiments’) with consideration and deduction of all coupled reactions in the data processing. All these aspects have been reviewed in great detail by Grossoehme, et al [49,50]. In short, the fundamental principles and strategies in handling metal-binding (equation 3) and ligand competition (equations 7a and 7b) discussed above in sections ‘Determination of metal-protein affinities via direct metal titration’ to ‘Design and optimisation of ligand or inter-metal competition experiments’ as well as the controls required in section ‘Key controls’ discussed later all apply equally to the ITC analysis. In reality, ITC may not detect equation 3 purely and thus the direct experimental values, *K*_ITC_ and Δ*H*_ITC_, can only be taken as the condition-dependent *apparent* values which must be converted to pH-dependent conditional values, *K*_D(P)_ and Δ*H^o^* in most cases (see equations 6b, 8a, 8b). In addition, ITC may have difficulty in handling slow kinetic processes. Lack of these considerations and corrections are the major sources of discrepancy in metal-binding ITC and have led to reports of many widely dispersed apparent ^a^*K*_D_ values in the literature, as highlighted in ref [51]. A recent study of the reduction thermodynamics of blue copper proteins provides an excellent example on how to undertake ITC analysis correctly [22].

### Spectroscopic analysis

This method is so far the most commonly used technique in metal–protein affinity determination. It offers an obvious advantage of being able to detect and quantify at least one species in the competition equilibrium directly without disturbing the established equilibrium based on a characteristic spectroscopic readout of that species in the system. The spectroscopic readout may include signal derived from UV–visible absorbance, fluorescence, circular dichroism, electron paramagnetic resonance (EPR) or nuclear magnetic resonance (NMR) [11,43]. The readout from a single species is usually enough for quantification of the binding equilibrium such as equations 3, 7a or 7b but it is sometimes possible, and may increase robustness, to detect multiple species in equilibrium simultaneously [52]. In the next section, we review in detail the application of some commonly used spectroscopic probes in metal–protein affinity determination.

## Spectroscopic probes as competing ligands and affinity standards

Spectroscopic probes based on solution absorbance and fluorescence are most widely used. In some cases, metal-binding may elicit a response from the protein itself: Some common examples include LMCT or metal d–d transitions originating from the metal–protein interaction [9,53,54]; and changes in native protein fluorescence, due to fluorescence quenching by paramagnetic metal ions, fluorescence enhancement by direct metal-fluorophore interaction, or protein conformational change as a result of metal-binding [41,55,56]. In such cases the protein itself or its metal complex may act as the detection probe and any metal ligand with matching or weaker affinity, provided that it does not interfere with the probe signalling, may be employed as a metal competitor. A large number of classic metal chelators with well-characterised affinities, such as Egta and Edta, are readily available for this purpose [11,15]. However, where the metal–protein interaction itself cannot be detected directly, a competing ligand that can report the metal-binding event (such as a spectroscopic probe) is a convenient tool. While many spectroscopic metal probes have been reported [11,12,57–59], only a handful are both robustly characterised with well-documented metal-binding affinities and readily available either commercially or via simple laboratory synthesis procedures. These factors are key to enabling their widespread practical applications. Selected examples of such probes for common transition metal ions (including Mn(II), Fe(II), Co(II), Ni(II), Cu(I), Cu(II) and Zn(II)) are given below. Their structures are shown in Figure 4 and their key properties are summarised in Tables 3 and 4.

### Metal probe ligands that form 1 : 1 ML complexes

#### General features

The ML-type probes described herein generally detect metal-binding via a change in the spectroscopic properties (absorbance and/or fluorescence) of the probe ligands themselves upon interaction with metal ions, thus they are mostly turn-off probes (in section ‘Other probes’ we also briefly describe several highly sensitive turn-on fluorescent and chromophoric probes that have been developed recently). To generate a best-fit *K*_D(P)_ from the experimental data, formulae may be derived from equation 7a with incorporation of related mass balances and can be tailored for different experimental procedures [19]. Equations 13a and 13b are two examples that can be conveniently employed where [P]_tot_ or [M]_tot_, respectively, are varied during the experiment. The experimental data may be fitted equivalently with available programs such as Dyanfit [60].

(13a) $$[\text{P}{]}_{\mathrm{tot}}\;=\;[\text{M}{]}_{\mathrm{tot}}-[\text{ML}]\;+\;\;\frac{K_{D(P)}}{K_{D(L)}}\;\left(\frac{{\left[\text{M}\right]}_{\mathrm{tot}}}{\left[\text{ML}\right]}-1\right)({\left[\text{L}\right]}_{\mathrm{tot}}-\;[\text{ML}])$$

(13b) $$[\text{M}{]}_{\mathrm{tot}}\;=\;\frac{{\left[\text{P}\right]}_{\mathrm{tot}}\;[\text{ML}]}{[\text{ML}]\;+\;\frac{K_{D(P)}}{K_{D(L)}}\;({\left[\text{L}\right]}_{\mathrm{tot}}\;-\;[\text{ML}])}\;\;+\;\;[\text{ML}]$$

#### Fura-2

The fluorescent probe fura-2 (Figure 4) forms 1 : 1 complexes with numerous divalent transition metal ions. Paramagnetic Mn(II) and Co(II) strongly quench the fluorescence signal [61,62] while diamagnetic Zn(II) blue-shifts the fluorescence excitation spectrum [61] and the binding affinities have been determined for each of these metal ions (Table 3). Fura-2 has proven particularly useful in the determination of Co(II)-binding affinities, including Co(II)-sensing transcription factors from *Escherichia coli* [63], Synechocystis [64], and *S. typhimurium* [34] and the Co(II) chelatase, CbiK, for the anaerobic vitamin B_12_ pathway in *S. typhimurium* [5]. Spectroscopic responses for Fe(II) [61] and Ni(II) [62] have also been reported and a robust characterisation of their binding affinities could further extend the applications of fura-2.

#### Mag-fura-2

Mag-fura-2 (Mf2, Figure 4) forms 1 : 1 metal complexes with characterised affinities for a variety of different ions (Table 3), making it a convenient and versatile probe. Metal-binding can be followed by changes in either absorbance or fluorescence spectra [65–67]. The apo-probe displays an absorbance maxima at ∼366 nm which blue-shifts to ∼325 nm upon binding numerous metal ions (including Mn(II), Fe(II), Co(II), Ni(II), Zn(II); see Figure 2A and Table 3) and the change in absorbance at either wavelength can be quantitatively correlated to the concentration of the metal-bound probe: extinction coefficients, ε, for the apo- and metal-bound probe are of the order of ∼10^4 ^M^–1^ cm^–1^ at the respective maxima [5,66] and the precise differences in ε at each wavelength, i.e. the Δ*ε* values associated with metal-binding, can be quantified by a control titrations of Mf2 with the metal of interest under bespoke experimental conditions (i.e. pH, [salt]; see section ‘Non-competitive controls, medium pH and buffers’). Alternatively, changes in fluorescence spectra upon metal-binding may be monitored: metal-binding typically leads to a decrease in Mf2 fluorescence (at ∼500 nm) when the excitation wavelength is set to ∼370 nm [67]. Mf2 has been widely used to quantify *K*_D_ values in the µM to nM range including various metal affinities (Mn(II), Co(II), Ni(II), Zn(II), Cd(II)) of the manganese transport regulator, MntR, from *Bacillus subtilis* [67]; affinity of the *E. coli* transcriptional regulator, RcnR, for one of its cognate metals, Ni(II) [63]; and affinities of *S. typhimurium* CbiK for non-cognate metals (Mn(II), Fe(II), Ni(II), Zn(II)) [5]. Mf2 has also been applied to define the stoichiometries of metalloproteins with tighter affinities, such as the human proteins calprotectin [68] and S100A12 [69] with sub-nM affinities for Zn(II).

#### Quin-2

Quin-2 (Figure 4) forms a 1 : 1 complex with Zn(II) ions at pH 7.0 and binding can be monitored via the UV-difference spectra [70], however, the signal change in the UV region (at *λ *∼ 265 nm, see Table 3) is susceptible to interference from competing proteins (via absorbance of aromatic amino acids) and from cofactors (e.g. nucleotides) which are sometimes present in competition experiments. Interfering factors should be taken into consideration in the experimental design and analysis, with appropriate controls carried out. The tight affinity of quin-2 has proven useful for quantifying binding affinities of native Zn(II) proteins where Mf2 is too weak to impose an effective competition, such as the Zn(II)-sensors ZntR and Zur from *S. typhimurium* [34] and AdcR from *S. pneumoniae* [71].

#### Dansylated peptides (DP1–4)

A series of peptide probes, each conjugated to a fluorescent dansyl moiety (*λ*_ex _∼ 330 nm; λ_em_ ∼ 550 nm; Table 3) via the side-chain amine of a lysine residue (Figure 4), respond to Cu(II)-binding through fluorescence quenching [19]. The four different peptide sequences in DP1 to DP4 bind Cu(II) with increasing affinities and together can be used to determine Cu(II) *K*_D_ from µM to fM range at biological pH (see Figure 5). Probe responses and affinities across a wider pH range (6.2–9.2) have also been characterised [19]. The peptides, which can be synthesised in house or purchased from commercial companies, have been used to determine Cu(II) affinities of the extracellular domains of the human amyloid precursor protein [72], native and mutated amyloid β peptide sequences [73], and several variants of the high-affinity Cu(II) chaperone, CopC, from *P. fluorescens* (see examples in Figure 2D–F) [35,74].

## 

### Metal probe ligands that form 1 : 2 ML_2_ complexes

#### General features

Several probe ligands in common application form ML_2_-type chromophoric complexes that can be quantified based on their solution absorbance at a particular wavelength in visible region where the probe ligands have negligible or significantly weaker absorbance (Table 4, see Figure 3(a,e,i) for selected examples). Consequently, these ML_2_ complexes may be considered broadly as turn-on probes and are very flexible and diverse in their applications (Table 1), as demonstrated by the several examples given in Figure 3 and Table 2. To generate a best-fit *K*_D(P)_ from the experimental data, formulae may be derived from equation 7b with incorporation of related mass balances for different experimental procedures [11,33]. Equations 14a–14c are examples that can be conveniently employed where [P]_tot_, [L]_tot_ or [M]_tot_, respectively, are varied during the experiment. The experimental data may be fitted equivalently with available programs such as Dyanfit [60].

(14a) $$\left[\text{P}{]}_{\mathrm{tot}}\;=\;[\text{M}{]}_{\mathrm{tot}}-\;[\text{M}{\text{L}}_{2}]+K_{D}\;\beta_{2}{\left(\frac{{\left[\text{L}\right]}_{\mathrm{tot}}}{\left[\text{M}{\text{L}}_{2}\right]}\;\text{–}\;2\right)}^{2}[\text{M}{\text{L}}_{2}]({\left[\text{M}\right]}_{\mathrm{tot}}-\;[\text{M}{\text{L}}_{2}]\right)$$

(14b) $$[\text{L}{]}_{\mathrm{tot}}\;=2[\text{M}{\text{L}}_{2}]+{\left(\frac{\left({\left[\text{P}\right]}_{\mathrm{tot}}\;-\;{\left[\text{M}\right]}_{\mathrm{tot}}\;+\;[\text{M}{\text{L}}_{2}])[\text{M}{\text{L}}_{2}\right]}{K_{D(P)\;}\beta_{2}({\left[\text{M}\right]}_{\mathrm{tot}}\;-\;[\text{M}{\text{L}}_{2}])}\right)}^{1/2}$$

(14c) $$[\text{M}{]}_{\mathrm{tot}}\;=[\text{M}{\text{L}}_{2}]+\frac{{\left[\text{P}\right]}_{\mathrm{tot}}\;[\text{M}{\text{L}}_{2}]}{[\text{M}{\text{L}}_{2}]\;+\;K_{D(P)\;}\beta_{2}\;{\left({\left[\text{L}\right]}_{\mathrm{tot}}\;-\;2[\text{M}{\text{L}}_{2}]\right)}^{2}}$$

#### Par

**Figure 6.**
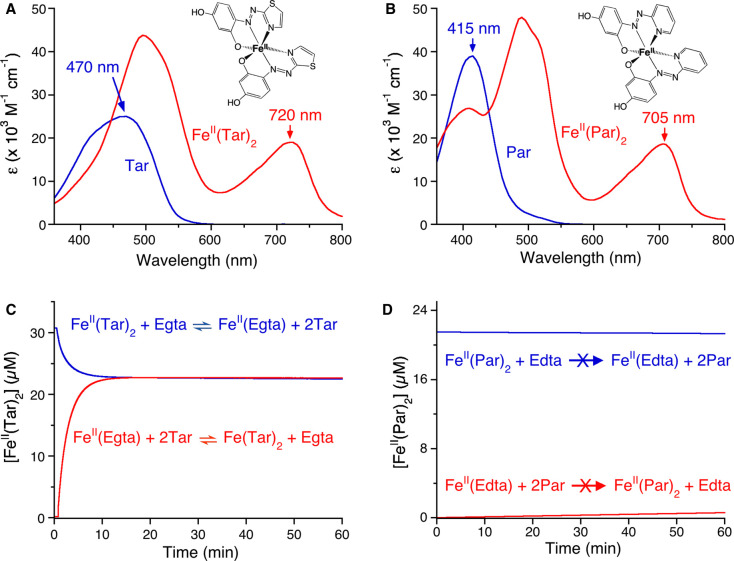
Fe(II) probes Fe^II^(Tar)_2_ and Fe^II^(Par)_2_ and their different exchange kinetics with Egta. (**a**,**b**) Solution spectra for the probes and the probe ligands in Mops buffer (50 mM, pH 7.2, 100 mM NaCl). Inset: proposed molecular structure of a geometric isomer of the respective probe; (**c**) the exchange kinetics between Tar (72 µM) and Egta (220 µM) for Fe(II) (30.8 µM) in two opposite directions in a mixing cell followed by A (720 nm) for Fe^II^(Tar)_2_; (**d**) The exchange kinetics between Par (72 µM) and Egta (36 µM or 3.6 mM) for Fe(II) (30 µM) in two opposite directions in a mixing cell followed by A (705 nm) for Fe^II^(Par)_2_. The data were adapted from ref. [78].

The chromophoric ligand 4-(2-pyridylazo)resourcinol (Par; Figure 4) is commonly used to probe zinc-binding, which produces a red-shift (and visible colour change) in the ligand absorbance. At neutral pH, the Zn^II^(Par)_2_ complex has an intense absorbance peak at ∼495 nm with an extinction coefficient around 60 times that of the ligand alone [28,75]; thus the concentration of the Zn^II^(Par)_2_ complex can be sensitively quantified from the absorbance change (with respect to the ligand only solution; see Table 4). A complicating factor is that Par forms both 1 : 1 and 1 : 2 complexes at pH 7.0, with equilibrium favouring the 1 : 1 complex in the presence of excess Zn(II) and the 1 : 2 complex in the presence of excess ligand [27,75]. For quantitative experiments, Zn(II) can be forced to coordinate exclusively via the 1 : 2 complex (>95% as given by the relationship [ML_2_]/[ML] = *K*_A2_[L], see ref. [11]; with *K*_A2_ = 5.5 × 10^5 ^M^−1^ for Par at pH 7.0, see refs. [75,76]) by maintaining a free (uncomplexed) Par concentration of >35 μM, in which case the spectroscopic and binding properties specific to the 1 : 2 complex (Table 4) can be used reliably: Such experimental setups have been used to quantify Zn(II)-binding to the *Synechococcus* metalloregulatory protein SmtB [53], the metal-binding domains of Zn(II)-transporting P_1B_-type ATPases (HMA2 and HMA4) from *Arabidopsis thaliana* [28,77], and to Zn(II)-finger peptides [27]. Notably, both the spectroscopic and metal-binding properties of Par are highly pH-dependent with a combination of different charge species present at physiological pH [27], thus particular care should be taken with pH control. Par also responds to several other divalent metal ions (including Fe(II), Co(II) and Ni(II) which each display 1 : 2 stoichiometry for metal : ligand binding) [27,78,79] and has been used, for example, to quantify Ni(II) release from proteins [80,81], but reported affinities determined in 50% dioxane/water solution [76] may differ in aqueous buffers. Slow ligand-exchange observed for Fe(II) complexes makes Par unsuitable as an equilibrium probe for Fe(II) (see a selected example in Figure 6) [78].

#### Tar

The chromophoric ligand 4-(2′-thiazolylazo)-resorcinol (Tar) is structurally similar to Par (Figure 4) and the free ligand displays a characteristic absorbance at ∼470 nm (Figure 6A). Tar forms 1 : 2 complexes with a variety of divalent metal ions at physiological pH, typically accompanied by a red-shift in the absorbance spectrum [6,78,82]. Fe(II)-binding produces an additional unique absorbance peak at 720 nm which is not observed for either the metal-free ligand or upon Fe(III)-binding to the ligand (although Fe(III) also displays a tight affinity; see Table 4). The Fe^II^(Tar)_2_ complex undergoes relatively fast ligand exchange with many other Fe(II) binders (an example is shown in Figure 6C) and thus Tar has been used as an effective probe to quantify enzyme-mediated Fe(II) oxidation [78]. Affinities for Fe(II) [78] and Ni(II) [6] have been quantified under physiological conditions (Table 4): For both metals, Tar can be used to quantify binding interactions which are too tight to be determined by Mf2 (Figure 5). Like Par, metal-binding properties of Tar (including spectroscopic responses and affinities) are pH-dependent, due to heterogeneous protonation/deprotonation of the benzenediol hydroxyl groups over physiological pH ranges [78].

#### Fs, Fz, Bca and Bcs

Ferrene S (Fs), ferrozine (Fz), bicinchoninic acid (Bca) and bathocuproine disulfonate (Bcs) comprise a complementary set of Cu(I)-binding probe ligands that have been robustly characterised and together can be used to determine Cu(I) affinities from nM to zeptomolar (zM) range [8,33,38]. Each forms a ML_2_-type Cu(I) complex with visible absorbance at wavelengths where the corresponding free ligands are spectroscopically silent (Figure 3A,E,I). These ligands can also bind Cu(II) but with weaker affinities and consequently, the respective Cu(I)-probe complexes can be readily prepared *in situ* by reaction of Cu(II) salts with an excess of each ligand in the presence of a suitable reductant (e.g. NH_2_OH or ascorbic acid with a combination of both optimal for Fs, see ref. [33]). Other than the weak affinity probe Cu^I^(Fs)_2_ which is modestly air-sensitive and must be handled under strict anaerobic conditions with excess reductant, the other three probes of higher affinities are more air-stable and may be handled routinely in N_2_-purged buffers in the presence of reductant NH_2_OH [8,33]. Due to the low p*K*_a_ values of coordinating atoms, metal-binding affinities for Fs, Fz and Bca are pH-independent at pH > 5 and Bcs (p*K*_a_ ∼ 5.7) is pH-independent at pH > 7 [8,33]. The set of probes have been used to determine a broad spectrum of Cu(I)-binding affinities from Methanobactins peptides with zM affinities (*K*_D _< 10^−20 ^M at pH 8.0) to the human amyloid β peptide sequence (*K*_D_ = 10^−10.4 ^M at pH 7.4 for Aβ1–16) and the D2 domain of amyloid precursor-like protein from *Caenorhabditis elegans* (*K*_D_ = 10^−8.6 ^M at pH 7.0) [25,38,83]. In fact, considering the case of the application of Cu^I^(Bca)_2_ to CopK [36], an application window spanning almost 10 orders of magnitude of affinities has been reached with Cu^I^(Bca)_2_ and Cu^I^(Bcs)_2_ alone. However, Cu^I^(Fs)_2_ and Cu^I^(Fz)_2_ are more suitable probes for protein targets with the Cu(I) affinities in the *K*_D(P)_ ≥ pM range such as for the Aβ peptides and related protein domains derived from the human amyloid precursor protein [25,84].

### Nature of detection probes and their ranges of application

Summarised in Figure 5 are the estimated affinity ranges of protein *K*_D(P)_ for several bio-essential first-row transition metals that may be determined reliably by the spectroscopic probes described above. The estimations are based on typical experimental designs detailed in the figure legend. However, as discussed in section ‘Design and optimisation of ligand or inter-metal competition experiments’, these ranges may be extended significantly by optimising the relative concentrations of each individual component including metal, protein and/or the competing ligand, provided that the detection sensitivity is not compromised with such optimisation. The application of turn-off probes fura-2, Mf2, quin-2 and DP peptides, which detect changes in the readout of the metal-free ligand upon metal-binding (see Table 3), can be extended to weaker affinity ranges by employing an excess of protein target, but not to much tighter affinity ranges since an excess of these metal-free probe ligands would compromise their detection sensitivities (see section ‘Turn-off probes and their restricted application windows’). In contrast, the turn-on probes Cu^I^(Fs)_2_, Cu^I^(Fz)_2_, Cu^I^(Bca)_2_, Cu^I^(Bcs)_2_ and Fe^II^(Tar)_2_, which detect metal-complex formation directly without interference from the free ligands, can be more flexibly extended to either weaker or tighter affinity ranges without interfering with the measured readout (see examples in Figure 3). Probes including Zn^II^(Par)_2_, Ni^II^(Tar)_2_ and Cu^I^(Bca)_2_ (using *λ *∼ 358 nm, see Table 4), which sensitively detect their metal-bound forms but with a residual signal from the free ligands, can be extended to somewhat tighter affinities but there is a limit to the concentration of free ligand that may be employed without excessive interference. An illustration of how a probe application range can be extended, using Cu^I^(Bcs)_2_ as an example, is shown in Figure 5.

### Other probes

Several well-characterised and commercially available probe ligands, while not obviously superior, can be employed as viable alternatives to those described above. For example, *K*_D_ values of the ML-type fluorescent indicators FluoZin-3 and Newport Green have been determined for multiple transition metal ions and are typically in the µM–nM range [85]; the absorbance probe Zincon forms ML complexes with Zn(II) and Cu(II) with affinities in µM and fM ranges, respectively [86]; and the ML_2_-type probe ligand Zinquin, which detects Zn(II) binding via absorbance or fluorescence, is well-matched for determining Zn(II) affinities in the nM range [87].

There are also many recently developed metal probes which can be used for protein affinity determinations and, indeed, may offer advantages for some applications. These include: a variety of highly sensitive, turn-on, ML-type Cu(I) probes such as the CS and CTAP series whose weak fluorescence, corresponding to the ligands alone, is dramatically enhanced (tens to hundreds of fold) in response to Cu(I)-binding [12,58]; a water-soluble bis(thiosemicarbazone) ligand that forms a 1 : 1 complex with Zn(II) that absorbs intensively in the visible region and was used as a sensitive chromophoric Zn(II) probe to quantify Zn(II) binding in proteins with nM affinities [88]; and a quinoline-based fluorescent sensor with fM-range affinity for Zn(II) [89], which could be applied to studies of tight Zn(II)-protein interactions beyond the application ranges of Par and quin-2. These ML-type probes may offer an advantage in avoiding formation of stable ternary complexes in rare cases where this might be a problem for the ML_2_-type probes such as a recently reported case in our study of Cu(I)-binding to a protein E2 domain of amyloid precursor protein [84]. However, these highly sensitive ML-type probes are not currently widely available.

## Non-competitive experiments for determination of metal–protein stoichiometry

Metal–protein binding stoichiometry is another important parameter for characterisation of metal–protein interactions and is also a key step in building a correct binding model for reliable affinity evaluation. In contrast with the discussion above, determination of metal–protein stoichiometry requires an experimental condition allowing (essentially) stoichiometric metal-binding to the targeting metal site(s). This is commonly determined by metal saturation of protein, followed by removal of excess metal, for example via co-migration of the metal–protein complex through a gel-filtration matrix, and then conducting separate metal and protein analyses [5,28,55]. However, some metal ions bound in proteins (such as Cu^+^), although possessing high affinities to many metal sites, are kinetically labile and may not survive the gel-filtration elution. In addition, some proteins may possess multiple metal sites (including non-native site(s)) with different affinities. Metal saturation of all these sites may induce protein denaturing and precipitation.

Direct characterisation in solution with spectroscopic probes proves to be an attractive and efficient alternative approach. The non-competitive metal-binding experiments presented in Figures 2 and 3 are examples. The experiment may be conducted either by titration of metal ions into a mixture containing a non-competitive weak affinity probe and a target protein of much stronger affinity (e.g. Figures 2B,E) or by titration of the target protein into a metal–probe complex which enables non-competitive metal-transfer from the probe to the targeting metal site(s) (e.g. Figure 3B,F,J). Of course, if the protein itself can act as a detection probe for metal-binding, the quantification may be undertaken by direct metal titration in the absence of other controlling ligands, as suggested by the case of *K*_D _< 0.05 [P]_tot_ in Figure 1A (see practical examples in refs. [5,6,34,41,55,56]). However, a bonus of competition with a metal probe is that the metal availability for a non-competitive metal-transfer to a specific protein targeting site may be controlled to distinguish *bona-fide* functional metal-binding from adventitious non-functional binding. This is demonstrated for hGrx1 in Figure 3F: as the Bca/Cu ratio was increased, progressively limiting the available [Cu_aq_^+^], hGrx1 was shown to bind multiple Cu(I) ions with *K*_D(P) _< 10^−13 ^M but only one Cu(I) with *K*_D(P) _< 10^−14 ^M (Table 2) [37].

## Key controls

This section outlines the key practical considerations and control experiments that are important for reliable *K*_D_ determinations. Table 5 provides a practical ‘checklist’ for avoiding the most common pitfalls.

**Table 5.** Checklist for avoiding pitfalls in metal-binding experiments

Potential pitfalls	Possible solutions
Is protein *apo*?	Proteins may acquire metals *in vivo* during expression or *in vitro* during isolation (e.g. from buffers, salts, IMAC resins) which may be removed by incubation of protein with a strong metal chelator (e.g. EDTA) before the final purification step.
Are metal ions and protein cysteinyl ligands fully reduced?	In studies that involve redox-active metal ions (e.g. Fe^II^ or Cu^I^) or sulfhydryl groups of protein cysteines that act as metal ligands (both sensitive to oxidation), it is essential to maintain reducing conditions during the metal-binding assay. Proteins may be purified with reductant(s) such as DTT (and related thiols) or TCEP but, these reductants are strong metal chelators [91,104] and thus the purified proteins should ideally be transferred into an anaerobic chamber and separated via buffer-exchange into a deoxygenated buffer unless the reductants are demonstrated to have no interference with the downstream metal-binding experiments. Metals (ideally prepared in anaerobic solutions) should also be completely reduced under the experimental conditions, with [metal]_red_ quantified by reaction with excess ligand (e.g. Bca, Bcs for Cu^I^ [8]; Fz for Fe^II^ [105]). Ellman's reagent can be used to quantify reduced protein thiols under denaturing conditions [106,107].
Are probe and metal concentrations correct?	*Apo*-probes can be quantified via absorbance using known extinction coefficients or by titration of probe with a calibrated metal stock (the latter is strongly recommended). Metal concentrations can be determined by AAS, ICP-MS, or simply by titration of metal into a calibrated probe solution.
Are buffers metal-free?	Many buffers and salts contain trace metals. Glassware used for preparation of solutions can also contain metal contamination. For steps following removal of chelators (see above) select high-grade buffers with certified low levels of trace metal contamination. Alternatively, chelex-resins can be used to remove metal ions from reaction buffers and the metal-free solutions can be eluted into (i) glassware that has been washed with nitric acid (4% (v/v), overnight); or (ii) directly into clean plasticware.
Are buffer components competing for metal-binding?	While strong metal chelators must be eliminated from metal-binding experiments, many buffers (e.g. Tris, phosphate) and salts (e.g. Cl^−^) display weak metal affinities and may contribute to metal speciation if present at high (millimolar) concentrations (e.g. millimolar [Cl^−^] can compete with Fs or Fz for binding Cu(I), see ref. [33]). A control metal titration of a probe-only solution can establish the reliability of the probe response and the potential contributions of buffers and salts (see section ‘Non-competitive controls, medium pH and buffers’). Non-coordinating buffers are available [95] and are preferred, especially for weak metal-binding experiments.
Are purification tags interfering?	Purification tags with comparable metal affinities to the protein sites interfere with downstream experiments and thus must be cleaved post-purification. However, non-competing purification tags can be retained (see Figure 2F and ref. [35]).
Is pH controlled?	Metal affinities and spectroscopic properties of probe ligands can be highly pH-dependent. Metals, ligands and reductants can influence buffer pH. Reaction solutions should be carefully monitored to ensure that pH is unchanged throughout the experiment. Where the competing ligand affinities are reported as ‘absolute’ formation constants, their conditional affinities at the experimental pH must be used in calculations [11].
Is competition at thermodynamic equilibrium?	Equilibration time for metal exchange between ligand competitors must be determined on a case-by-case basis. Where metal exchange is fast, competition experiments can be carried out as continuous titrations; but some competitions required prolonged incubation to reach equilibrium (see section ‘Kinetic controls’ and Figure 6).
Are stable ternary complexes present?	The formation of stable ternary complexes prevents meaningful quantification of metal affinities via equation 7a/b. Several control experiments to confirm or disprove their presence have been described (see section ‘Ternary complexes’ and ref. [84]).
Are equilibrium measurements reliable?	Ensure that there are some competitive data points where the probe responses lie within the 20–80% range of the control response in the absence of competition (see sections ‘Determination of metal–protein affinities via competition experiments’ and ‘Design and optimisation of ligand or inter-metal competition experiments’). This may be confirmed by control experiments in the absence of competition or tested by simulations of binding curves modelling responses for 10-fold tighter and weaker protein affinities (see examples in Figure 2).

### Sample quality

Measurements of metal affinity require accurate quantification of the equilibrium concentrations of each reaction component (M, L and P), and this is especially important for the limiting component(s). Proteins must be pure, metal-free, fully reduced (if they contain air-sensitive cysteinyl thiol groups that act as metal ligands), accurately quantified, and free of non-native metal-coordinating tags (if their metal affinities are comparable to those of the targeting protein sites). This can be confirmed via a series of control experiments outlined in Table 5. Ligand and metal concentrations should be calibrated via spectroscopy and/or elemental analysis (Table 5). Reaction conditions must be strictly controlled to avoid metal contamination and to prevent oxidation of susceptible metal ions or ligands (see Table 5). Chelants and reductants can be used to remove contaminating metals and to maintain reducing conditions during protein purification but must not be present in the final assay solutions, unless it has been explicitly demonstrated that their presence has negligible impact on the metal-binding equilibria. For example, both DTT and TCEP are commonly used protectants for protein thiols but they are strong chelators for Cu(I) and cannot be present for most Cu(I) affinity determinations [8,90,91]. On the other hand, sodium dithionite is a very strong reductant (*E*_1/2_ = −0.66 V vs SHE) [92] and can reduce cysteinyl disulfide *in situ* rapidly, but dithionite itself and its oxidation products are not Cu(I) ligands. Consequently, dithionite has been used as a convenient *in situ* reductant for protein disulfide for Cu(I) affinity determination with Cu^I^(Bca)_2_ and Cu^I^(Bcs)_2_ probes [93,94], but dithionite is a strong reductant for some metal ligands such as Fs and Fz and is not compatible with the applications of these ligands [33].

### Non-competitive controls, medium pH and buffers

For any ligand competition experiment, a key control is the non-competitive titration of metal into probe under identical experimental conditions (i.e. pH buffer, salts). This serves to (i) define the metal-binding stoichiometry of the probe ligand; (ii) calibrate the probe response to metal under non-competing conditions (i.e. the control response); and (iii) confirm that metal ions bind stoichiometrically to the probe ligand under the experimental conditions without competition from adventitious ligands (such as buffers, salts, hydroxide; see section ‘Adventitious metal ligands and the apparent dissociation constant’). Examples of such titrations, defining the fluorescent responses of probes DP2 and DP4 to Cu(II) ions and the chromophoric response of probes Fz, Bca and Bcs to Cu(I) ions, are shown in Figures 2E,F and 3I: A defined turning point at saturating metal concentration ([metal] : [ligand] of 1 : 1 for Cu^II^-DP2, Cu^II^-DP4; and 1 : 2 for Cu^I^(Bcs)_2_, Cu^I^(Bca)_2_ and Cu^I^(Fz)_2_) confirmed negligible dissociation or competition from adventitious ligands under the experimental conditions. In contrast, the response of the (relatively weak) Cu(I) probe Cu^I^(Fs)_2_ is linear only up to a Cu(I) : Fs ratio of ∼0.4, thus an experimental condition with an excess of Fs (>2.5-fold), void of potential (even weak) Cu(I) or Cu(II) ligands or buffers (Cl^−^, MeCN, Tris, KPi), under strict reducing conditions must be imposed to ensure a reliable response of the Cu^I^(Fs)_2_ probe (Figure 3I) [33]. In addition, a buffer is required to maintain solution pH (which should be monitored, see Table 5) and may be selected to minimise adventitious metal coordination [95]. The buffer concentration may be varied relative to the probe concentration to evaluate and/or minimise its competition but must retain sufficient buffering capacity to ensure a consistent pH throughout the entire experiment.

### Kinetic controls

Metal–protein affinity is a thermodynamic parameter and, critically, the metal-exchange reaction between the two competing partners must have reached thermodynamic equilibrium in order for *K*_D(P)_ to be determined meaningfully. However, the metal-exchange kinetics could be very different between different ligand partners and may also vary with experimental conditions. The best way to estimate the minimal time required for a new reaction is to follow the same metal-exchange reaction starting from two opposite directions. Two such examples are given in Figure 6 [78]. Both Tar and Par are similar metal ligands which form similar chromophoric ML_2_-type probe complexes with Fe(II) (Figure 6A,B). The Fe(II)-exchange between Tar and Egta reached equilibrium in ∼15 min (Figure 6C). However, surprisingly, the Fe(II) bound to either Par or Egta undertook essentially no exchange between the two, even when the thermodynamic driving force was amplified by increasing [Egta] from tens of µM to mM scales (Figure 6D). Therefore, the timescale for metal-exchange between each pair of competing partners should be assessed on a case-by-case basis. For those reactions that can reach equilibrium rapidly (i.e. within seconds or minutes), a continuous titration may be performed with a short incubation interval between each titration point. For those reactions that are slow (i.e. hours or even days) to reach equilibrium, titrations should be performed by preparing a series of solutions to enable prolonged equilibrium time. The continuing titration method consumes minimal sample but is subject to error due to stepwise solution dilution, especially for those binding equilibria with unequal molar numbers of reactants and products that shift their equilibrium position with dilution (such as those cases in Figure 3). The series solution method avoids dilution error and allows sufficient time to reach the metal-exchange equilibrium but consumes more sample.

### Ternary complexes

Transfer of metal ions between the competing partners in ligand competitions of equation 7a or 7b are frequently mediated via formation of transient protein–metal–ligand ternary complexes, PML. As long as such complexes are short-lived with their static concentrations contributing negligibly to the total speciation of the system, the validity of equation 7a or 7b will not be affected. However, if such PML complexes are stable and make a significant contribution to the total speciation of the system, as reported in many cases [84,96–98], they invalidate the relationships of equation 7a or 7b and preclude a meaningful quantification of *K*_D(P)_. It is essential to be certain that this is not the case for affinity determination by ligand competition. During our study of Cu(I)-binding to the protein domain E2 of the amyloid precursor protein, we identified and isolated such a stable PML complex that could be readily dissociated under certain conditions [84]. A systematic study of this system allowed us to document several ‘tell-tale’ diagnostic signs of the formation of such stable ternary complexes, and to suggest a set of simple control experiments to confirm or disprove their presence in solution [84]. These include:

(i) Confirmation that the probe spectra are free of distortions since a stable PML complex accumulates and may develop distinct spectroscopic features.
(ii) Recovery of a full probe response at saturating metal concentrations (i.e. [M]_tot_ ≥ [P]_tot_ + [L]_tot_) since stable PML complexes can alter the probe response.
(iii) Derivation of a consistent *K*_D(P)_ from multiple experimental conditions, including different ratios of [P]_tot_/[L]_tot_ (e.g. Figure 3K,L) or a simple ‘dilution effect’ experiment for equilibrium 7b that, if free of stable PML complexes, will shift the equilibrium position of each component upon dilution to maintain a constant *K*_ex_ and thus *K*_D(P)_ (see Figure 3C,D,G), unless the reaction is essentially non-competitive (Figure 3B).
(iv) Quantification of a consistent *K*_D(P)_ via competition with multiple ligand affinity references such as the case of CopK with probes Cu^I^(Fs)_2_ and Cu^I^(Fz)_2_ and of hGrx1 with probes Cu^I^(Bca)_2_ and Cu^I^(Bcs)_2_ (Figure 3).

## Summary and concluding remarks

Metal ions, and the transition metals in particular, are indispensable structural and catalytic cofactors in about a third of all proteins but they are double edge swords and can be toxic if not processed correctly. The speciation of metal ions *in vivo*, vital to cell function, depends substantially on the thermodynamics of metal–protein interactions. The dissociation constant, *K*_D_, is a critical parameter quantifying the driving force of such interactions but can be challenging to determine experimentally. If a metal–protein interaction is sufficiently weak, i.e. its *K*_D_ value is not more than 20-fold below the experimental protein concentration, the corresponding metal–protein affinity may be determined via direct metal titration, provided that competitive metal-binding from adventitious ligands (buffers, salts, etc.) is either negligible or fully accountable with appropriate controls. However, in practice, this is usually difficult for proteins since the protein sidechains often make a substantial contribution to such adventitious metal-binding at µM free metal concentrations. In the majority of cases, a functional metal–protein interaction is too tight to allow measurable dissociation even at the lowest limit of an experimental protein concentration and a competition experiment becomes the gold standard for the *K*_D_ determination. Even tremendously small *K*_D_ values in the aM to zM range have been reliably determined with this method.

Numerous spectroscopic metal-binding probes have been developed as convenient ligand competitors. To ensure the reliability of an affinity determination, the control response of a chosen probe in the absence of competition must have sufficient sensitivity (allowing a good signal-to-noise ratio) and the competitive responses should be measurably distinct (ideally within 20–80% of the control response). The former may be optimised by adjusting the total probe concentration and the latter by adjusting the relative concentrations of the competing partners. However, the ability to optimise such experimental parameters depends critically on the nature of the chosen probe: A ‘turn-off’ probe detects spectroscopic changes to the metal-free probe ligand upon metal-binding and its detection sensitivity is restricted by the metal occupancy of the probe ligand, but a ‘turn-on’ probe detects the metal-bound species directly with little restriction on the concentration of the metal-free probe ligand and is thus more flexible and versatile in its application.

Common to all quantitative analysis, each key assay component must be of high quality with careful concentration calibration. Important specifically to ligand competition, key controls must be conducted to ensure that the metal-exchange has reached a stable thermodynamic equilibrium with negligible (if any) impact from potential ternary complexes. *K*_D_ values, if determined reliably and available for a wide variety of metal–protein interactions, will provide a solid foundation to better understand cellular mechanisms of metal homeostasis, including various metal pumps that may overcome unfavourable thermodynamic gradients of metal-flow, protein partnerships that may facilitate specific metal trafficking routes and metal-compartmentalisation that may isolate metal availabilities within specific destinations. After all, proteins have evolved sophisticated strategies to select different metals. A complete picture of these complex networks of metal speciation will allow valuable insights into how protein metalation is regulated in diverse biological contexts.

## Open Access

Open access for this article was enabled by the participation of Durham University in an all-inclusive *Read & Publish* pilot with Portland Press and the Biochemical Society under a transformative agreement with JISC.

## Abbreviations

CD: circular dichroism
ICP-MS: inductively coupled plasma mass spectrometry
ITC: isothermal titration calorimetry
LMCT: ligand to metal charge transfer
NTA: nitrilotriacetic acid
